# Thermokarst lake drainage halves the temperature sensitivity of CH_4_ release on the Qinghai-Tibet Plateau

**DOI:** 10.1038/s41467-025-57356-x

**Published:** 2025-02-26

**Authors:** Mei Mu, Cuicui Mu, Hebin Liu, Pengsi Lei, Yongqi Ge, Zhensong Zhou, Xiaoqing Peng, Tian Ma

**Affiliations:** 1 Key Laboratory of Western China’s Environmental Systems (Ministry of Education), College of Earth and Environmental Sciences, Observation and research station on Eco-Environment of Frozen Ground in the Qilian Mountains, Lanzhou University, Lanzhou, China; 2 State Key Laboratory of Cryospheric Science, Northwest Institute of Eco-Environment and Resources, Chinese Academy of Sciences, Lanzhou, China; 3 Academy of Plateau Science and Sustainability, Qinghai Normal University, Xining, China; 4 State Key Laboratory of Herbage Improvement and Grassland Agroecosystems, College of Pastoral Agriculture Science and Technology, Lanzhou University, Lanzhou, China

**Keywords:** Carbon cycle, Geochemistry

## Abstract

Thermokarst lakes as hot spots of methane (CH_4_) release are crucial for predicting permafrost carbon feedback to global warming. These lakes are suffering from serious drainage events, however, the impacts of lake drainage on CH_4_ release remain unclear. Here, synthesizing field drilling, incubation experiments, and carbon composition and microbial communities, we reveal the temperature sensitivities (Q_10_) and drivers of CH_4_ release from drainage-affected lakes on the Qinghai-Tibet Plateau. We find that cumulative CH_4_ release decreases with depth, where 0–30 cm-depth sediment accounts for 97% of the whole release. The Q_10_ of surface sediment is 2 to 4 times higher than deep layers, but roughly 56% lower than the non-drainage lakes. The response of CH_4_ release to warming is mainly driven by microbial communities (49.3%) and substrate availability (30.3%). Our study implies that drainage mitigates CH_4_ release from thermokarst lakes and sheds light on crucial processes for understanding permafrost carbon projections.

## Abstract (web summary)

Little is known about the impact of thermokarst lake drainage on CH_4_ release. Here, the authors find that thermokarst lake drainage halves the temperature sensitivity of CH_4_ release, revealing the role of microbial community and substrate availability in regulating CH_4_ release.

## Introduction

**Fig. 1:**
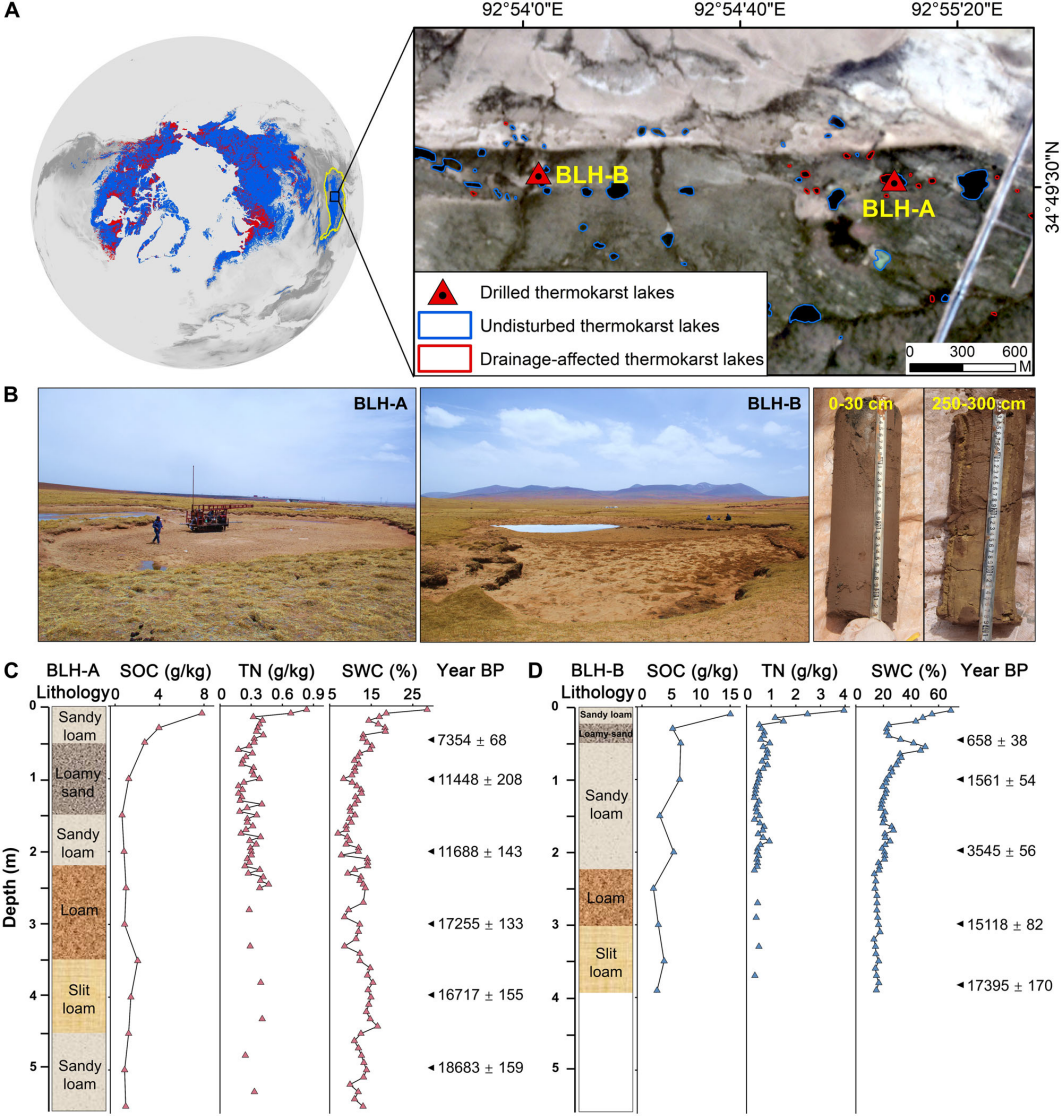
Surveys of drainage-affected thermokarst lakes and properties of deep sediments. **A** Study area with the spatial distribution of lake drainage events in the Northern Hemisphere permafrost regions^14^. The observed study area is located in the Beilu River region of QTP with the background of satellite imagery from PlanetScope 2021 (https://earth.esa.int/eogateway/missions/planetscope). The red colour represents the drainage-affected thermokarst lakes, and the blue represents the non-drainage lakes. The red triangles show the drilled thermokarst lakes at the BLH-A and BLH-B sites. **B** Field pictures of thermokarst lake BLH-A and BLH-B affected by drainage as well as the sampled deep sediment cores. **C**, **D** Characteristics of two deep lake cores with the distributions of soil organic carbon (SOC) content, total nitrogen (TN) content, sediment water content (SWC), and calibrated ^14^C dating and uncertainty (year BP) of SOC are shown on the right. Lithological units are identified by sediment texture. The BLH-A includes sandy loam, loamy sand, loam, and silt loam from top to deep layers. The BLH-B includes sandy loam, loamy sand, loam, and silt loam along the depth. Photos are taken by M.M. Source data are provided as a Source Data file.

Permafrost regions store ~30% of the world’s soil organic carbon in approximately 15% of the Northern Hemisphere land area^1,2^. Global warming causes the thawing of permafrost and accelerates the formation, expansion, and drainage (seasonal, intermittent, or permanent) of thermokarst lakes^3–5^. These lakes and drained lake basin systems cover more than 20% of the circumpolar Northern Hemisphere permafrost regions^3,6^. Thermokarst lakes formation and expansion increase methane (CH_4_) emission as accelerated permafrost thaw beneath and around lakes unlocks previously frozen sediments for microbial anaerobic decomposition^7,8^. Various studies have shown that thermokarst lakes serve as significant natural emission sources of CH_4_^7,9–11^, contributing 4.1–6.1 Tg CH_4_ per year to the atmosphere^12^. It was estimated that thermokarst lakes would release 30–60 billion tonnes of carbon into the atmosphere by 2300^13^, playing a crucial role in predicting permafrost carbon-climate feedback. Conversely, thermokarst lake drainage intensely alters hydrological dynamics^4^, potentially affecting CH_4_ release to the atmosphere^3^. In the past 40 years, over 35,000 lakes have suffered from drainage events in the northern permafrost regions, half of which are thermokarst lakes (Fig. 1)^14^. However, the impact degree of thermokarst lake drainage on CH_4_ release is poorly understood.

CH_4_ as a powerful greenhouse gas is produced in anaerobic environments, and drainage events can significantly change CH_4_ release from thermokarst lakes by influencing sediment moisture, carbon decomposability^15^, and vegetation type markedly^16–18^. Influenced by drainage events, microbial abundance and methanogenic communities of lake sediments had great changes^19,20^. Additionally, it was shown that sediment carbon composition such as mineral-associated organic carbon (MAOC) contents increases in the drainage process due to the protection of flocculation, sorption, and co-precipitation^21^. These influencing factors are the main determinants of CH_4_ release from thermokarst lakes^22–24^. Temperature sensitivity (Q_10_) represents a key parameter of biogeochemical models that reflects the response of carbon release to warming^25–27^. Quantifying the Q_10_ of CH_4_ release is thus critical to improving CH_4_ emissions assessments of thermokarst lakes and narrowing the uncertainty of permafrost carbon-climate feedback projections^28,29^. However, the Q_10_ of CH_4_ release and its drivers in the drainage-affected thermokarst lakes remain unclear, greatly hindering the accurate assessment of permafrost carbon feedback under forthcoming climate scenarios.

The Qinghai-Tibet plateau (QTP) is the largest mountain permafrost region globally, and its warming rate is about twice that of global warming^30^. The QTP permafrost region stores a large amount of soil carbon with 12.4–25.6 billion tonnes of organic carbon in the top 2 m of soil^31^. The QTP hosts approximately 161,300 thermokarst lakes covering a total area of ∼2800 km^2^ ^32^. Although rapid warming and consequent widespread permafrost thaw caused some expansion of thermokarst lakes^33^, drainage events of thermokarst lakes also emerged across the QTP over the past 40 years^34^. However, the impact of these lake drainages on CH_4_ release is poorly understood and is also not considered in current Earth System Models, which potentially results in a rise in the uncertainty of carbon-climate feedback in the alpine permafrost regions.

To fill the knowledge gap, we drilled two 5 m-depth sediment cores in the drainage-affected thermokarst lakes on the central QTP (Fig. 1). By combining carbon composition analyses, microbial high-throughput sequencing, and radioactivity dating techniques, we conducted an anaerobic experiment over 150-day durations to present the CH_4_ release in terms of vertical distribution, temperature sensitivity and driving mechanisms in the drainage-affected thermokarst lakes. Furthermore, by synthesizing the published Q_10_ data on CH_4_ release in the non-drainage thermokarst lakes in the same vegetation type^23^, we revealed the changes of Q_10_ of CH_4_ release suffering from lake drainage. Finally, we showed the multivariate effects of influencing factors on Q_10_ and relative importance using structural equation modelling (SEM) and hierarchical partitioning modelling. To our knowledge, this study first examines the dynamics of CH_4_ release in drainage-affected thermokarst lakes in the alpine permafrost regions.

## Results and discussion

### Substrate availability and microbial communities

**Fig. 2:**
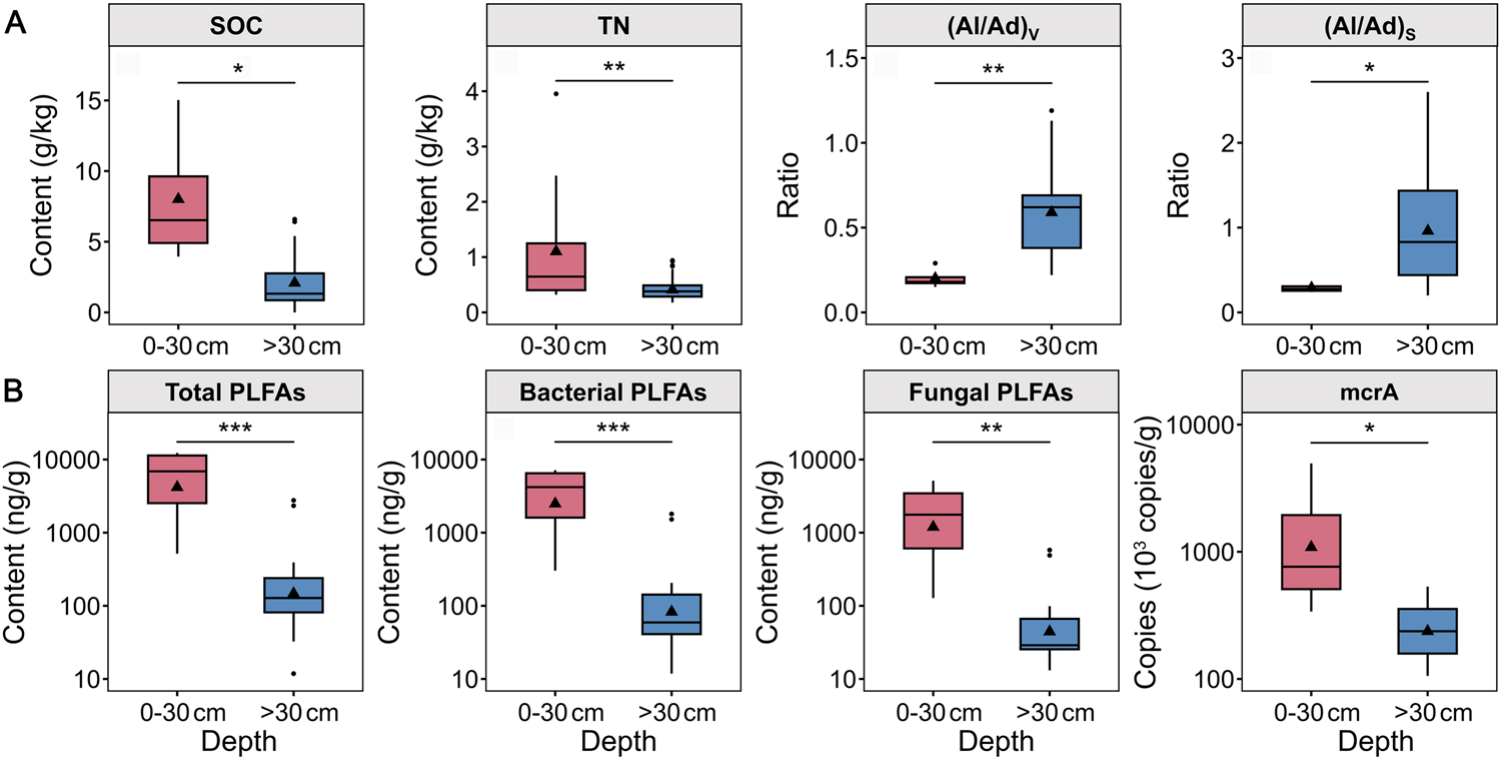
Substrate availability and microbial abundance in surface (0–30 cm) and deep sediments (>30 cm) of the thermokarst lakes. **A** The diagram shows the soil organic carbon (SOC), total nitrogen (TN), the ratio of acid to aldehyde forms of vanillyls ((Ad/Al)v), and the ratio of acid to aldehyde forms of syringyls ((Ad/Al)s). **B** depicts the microbial abundance of total, bacteria, and fungi, as well as the mcrA gene copies. Red and blue boxes indicate surface (0–30 cm) and deep sediments (>30 cm), respectively. The solid line and triangle in the box represent the median and mean of each dataset, respectively. The upper and lower ends of boxes denote the 0.25 and 0.75 percentiles, respectively. The upper and lower whisker caps indicate the 1.5 interquartile range of upper and lower quartile, respectively. Dots outside whiskers indicate outliers. *, ** and *** indicate significant differences at *p* < 0.05, *p* < 0.01, *p* < 0.001, respectively. Source data are provided as a Source Data file.

To reveal the vertical distribution of carbon composition and microbial communities, we conducted an analysis of multiple indices based on biochemical experiments (See methods). Sediment moisture content rapidly decreases along the depth profile and has the highest value of 14–69% in the surface 0–30 cm (Fig. 1C, D). Compared to saturated conditions in non-drainage thermokarst lakes, lake drainage significantly reduces sediment moisture contents and increases oxygen (O_2_) availability. The calibrated ages of soil organic carbon (SOC) of deep thermokarst lake sediments are from 7,354 ± 68 to 18,683 ± 159 years BP at BLH-A site (Fig. 1C) and 658 ± 38 to 17,395 ± 170 years BP at BLH-B site (Fig. 1D), respectively. The carbon ages of sediment from the two lakes increase gradually with depth, which indicates that surface sediment has a younger carbon age and shorter time of carbon turnover. The contents of SOC and total nitrogen (TN) decrease with depth in both drainage-affected thermokarst lakes (Fig. 1 C and D), and are significantly higher in the surface 0–30 cm layer than in the deeper sediments (>30 cm) (*p* < 0.01; Figs. 2A and S2). This phenomenon is consistent with the vertical distribution of SOC and TN in the QTP permafrost regions^35^. In contrast, the ratio of acid to aldehyde forms of vanillyl and syringyl monomers [(Ac/Al)_V_ and (Ac/Al)_S_] increases significantly with depth (Figure S1), and is significantly higher in deeper sediments (all *p* < 0.05; Fig. 2A). The ratio of SOC to TN (SOC/TN), particulate organic carbon to SOC (POC/SOC), and mineral-associated organic carbon to SOC (MAOC/SOC) have great variations with sediment depth (Fig. S1). Although these indices did not exhibit significant differences between surface and deeper layers (*p* > 0.05; Fig. S2), the ratios of POC/SOC and MAOC/SOC have an opposite trend. Taken together, our study demonstrates that higher substrate availability in surface sediment of thermokarst lakes is mainly dominated by POC compared to the deeper layers.

The abundances of all microbial groups, including bacterial, fungal, and actinomycetes PLFAs, significantly decrease with depth (Figs. S1 and 2B). The higher microbial abundance in surface 0–30 cm depth (all *p* < 0.01; Fig. 2B) could be attributed to higher moisture and substrate availability in surface sediment. Given the potential differences in methanogens and their metabolic pathways from different depth sediments, the functional gene abundance and community composition of methanogens were further analysed based on real-time PCR and high-throughput sequencing. The mcrA gene, which encodes a key enzyme in methanogenesis, serves as an indicator of methanogenic archaea abundance. We found that the functional gene abundance of methanogens decreases rapidly with depth (Fig. S1), with a significantly higher value in the surface than in the deeper layer (*p* < 0.05; Fig. 2B). This result indicated that there were greater potentials for CH_4_ production in surface sediments of these two drainage-affected thermokarst lakes. The large differences in methanogens abundance depends on the corresponding disparity of substrate and sediment environmental properties between the surface and deeper layer. Regarding the methanogenic community composition of sediment from drainage-affected thermokarst lakes, there were three dominant methanogenic orders, including *Methanomicrobiales*, *Methanobacteriales*, and *Methanosarcinales* (Fig. S3). This finding is consistent with results from non-drainage thermokarst lake sediments on the QTP^9^ and drainage-affected permafrost tundra in the Arctic^36^. The order composition diagrams showed that *Methanomicrobiales* and *Methanobacteriales* were the most abundant methanogenic orders, representing 88-99% of all methanogens. It has been reported that the orders *Methanomicrobiales* and *Methanobacteriales* take carbon dioxide (CO_2_) plus hydrogen (H_2_) as substrates to produce CH_4_^37^. The results reveal that the CH_4_ production pathway of drainage-affected thermokarst lakes can predominantly hydrogenotrophic, which is consistent with a recent study about non-drainage thermokarst lakes on the QTP based on stable carbon isotope (δ^13^C) of CH_4_ and CO_2_^9^. This is possibly attributed to sediment organic matter affected by waterlogging before lake drainage is not completely degraded, providing the substrates for CH_4_ production such as benzoate, CO_2_, and H_2_^38^.

### Temperature sensitivity of CH_4_ release

**Fig. 3:**
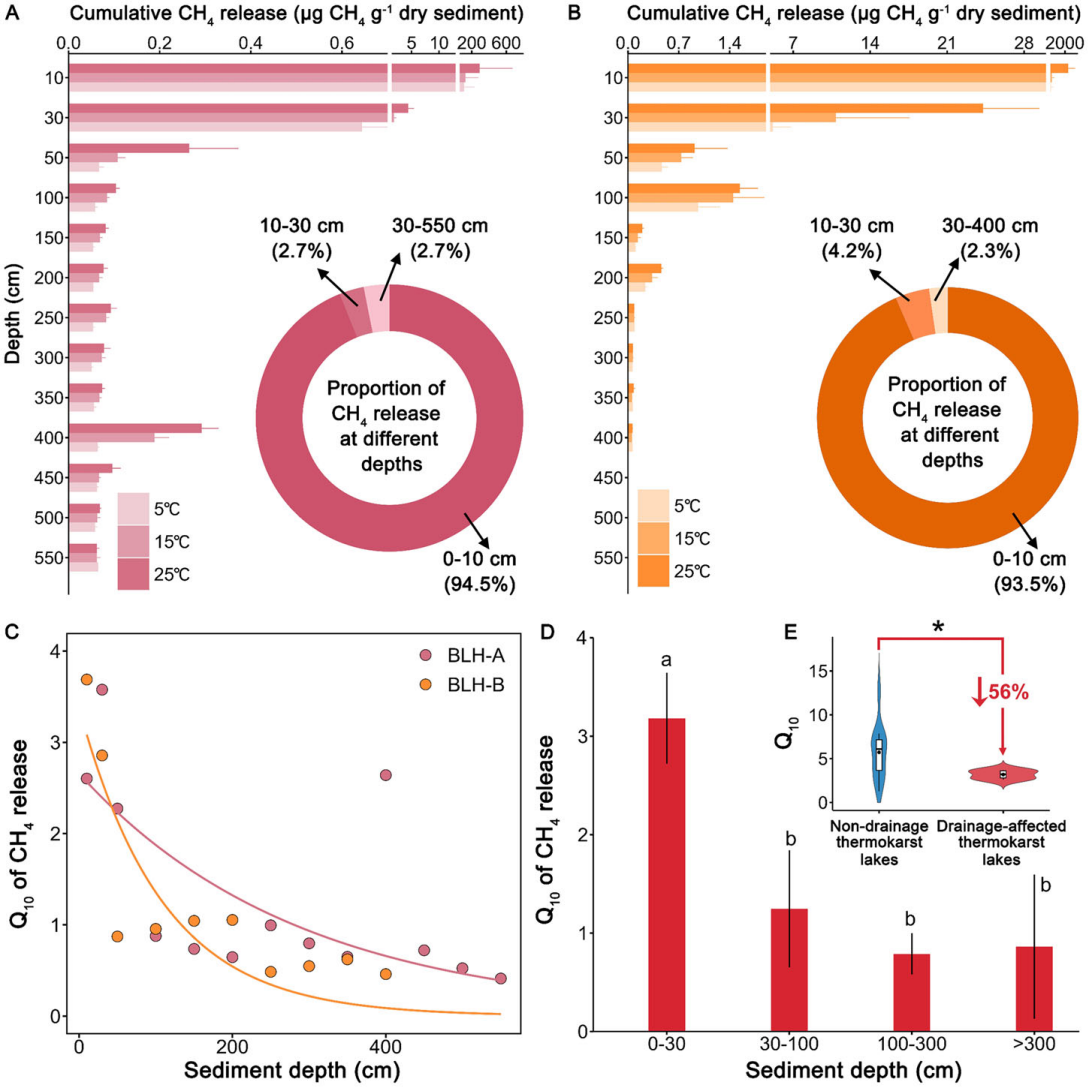
Cumulative CH_4_ release and its temperature sensitivity (Q_10_) of thermokarst lakes. **A**, **B** The bar charts show the cumulative CH_4_ release with different sediment depths at thermokarst lakes of BLH-A and BLH-B. The columns with different colours represent the CH_4_ release at the incubation temperatures of 5, 15, and 25 °C, respectively. Values are means ± standard errors (SE) (n = 4). The doughnut charts indicate the proportions of CH_4_ release from sediments at depths of 0–10 cm, 10–30 cm, and >30 cm. **C** The diagram shows the changes of Q_10_ with sediment depth in two thermokarst lakes affected by drainage. **D** The distribution of Q_10_ values of CH_4_ release at depths of 0–30 cm, 30–100 cm, 100–300 cm, and >300 cm from the drainage-affected thermokarst lakes. **E** The comparison of Q_10_ values of CH_4_ release in 0–30 cm layer between the drainage-affected thermokarst lakes and non-drainage thermokarst lakes on the QTP. The data from a publication^23^ and our unpublished data are shown in the supplementary materials. Values represent means ± SE. The solid line and black dots in the box represent the median and mean of each dataset, respectively. The upper and lower ends of boxes denote the 0.25 and 0.75 percentiles, respectively. The upper and lower whisker caps indicate the 1.5 interquartile range of upper and lower quartile, respectively. Source data are provided as a Source Data file.

To estimate the potential CH_4_ release and its Q_10_ in the sediment from drainage-affected thermokarst lakes, we conducted a long-term incubation experiment (See methods). The potential CH_4_ release rates decrease significantly with depth in thermokarst lakes of BLH-A and BLH-B, with the respective ranges of 0.002–13.50 and 0.001–65.70 μg CH_4_ g^−1^ dry sediment d^−1^ (Fig. S4). Similarly, cumulative CH_4_ release decreases significantly with depth, where 0–30 cm depth of sediment accounts for 97.2–97.7% of the whole-column CH_4_ release (Fig. 3A, B). This result showed that CH_4_ release of thermokarst lakes is mainly from the organic-rich surface lake sediments, which is consistent with a previous study that organic-rich mud facies accounted for 67% of whole-column CH_4_ production in the sediment core^39^. By contrast, the cumulative CH_4_ release was higher in BLH-B than in BLH-A, especially in the surface 0–100 cm, which may be due to large differences in moisture content, SOC and TN content, and microbial abundance in the sediment cores. Temperature increases accelerate the potential CH_4_ release with different sediment depths, especially in the 0–30 cm layer. We find that the Q_10_ of CH_4_ release decreases with sediment depth (Fig. 3C), with the ranges of 0.41–3.58 and 0.46–3.69 in thermokarst lake BLH-A and BLH-B, respectively (Fig. S5). The highest Q_10_ value occurs in the surface 0–30 cm of lake sediment (3.18 ± 0.46), which is remarkably higher than other depths (>30 cm) (all *p* < 0.001; Fig. 3D). These results corroborate the distribution of substrate availability, total microbial abundance and functional gene abundance of methanogens in the vertical profiles (Fig. 2). The results suggest that the Q_10_ of surface sediment in the drainage-affected thermokarst lakes is 2 to 4 times greater than deep layers (>30 cm), which provide essential parameters for permafrost carbon models.

Furthermore, to examine the effects of carbon ages on the potential CH_4_ release and its Q_10_, we analyzed the relationships of potential CH_4_ release and Q_10_ with carbon dating (See methods). The results illustrate that potential CH_4_ release and Q_10_ decreased nonlinearly with carbon dating increasing, which is more pronounced in BLH-B (Fig. S6). The highest CH_4_ release corresponds to the younger carbon in the vertical sediment profile, which is corroborated at larger study scales^40,41^. Actually, carbon dating serves as a proxy of organic matter reactivity, usually older carbon undergoes a longer period of microbial degradation, resulting in a lower reactivity than younger carbon^40^. Our finding reveals that younger carbon was a dominant source of CH_4_ production in the drainage-affected thermokarst lakes on the QTP, which is consistent with the field observations^9^.

To reveal the changes of Q_10_ affected by lake drainage, we conducted a preliminary comparison between the drainage-affected and non-drainage thermokarst lakes by integrating the currently published and our unpublished Q_10_ data on the QTP based on the similar incubation experiments (Table S2; See methods). Remarkably, compared with the non-drainage thermokarst lake (with an average Q_10_ value of 7.20 ± 5.76), the Q_10_ of CH_4_ release significantly declines in the drainage-affected thermokarst lakes (*p* < 0.05; Fig. 3E). This result ties well with previous studies, showing lower water table can cause a decrease in the temperature dependence of CH_4_ emissions in wetland ecosystems^42^. Our study quantifies the decrease of approximately 56% in the Q_10_ of CH_4_ release in the drainage-affected thermokarst lakes (Fig. 3E). This result suggests that low temperature sensitivity of drainage-affected thermokarst lakes is essential for assessing CH_4_ emissions from thermokarst lakes. There are two possible reasons for the low temperature sensitivity. First, thermokarst lake drainage dramatically alters oxygen conditions and hydrological^3^, threatening the survival of methanogens. On the one hand, thermokarst lake drainage exposes surface sediments to the atmosphere, resulting in the death of methanogens due to oxygen toxicity^43^. On the other hand, lake drainage reduces the water table depth and increases water stress on microorganisms, thereby decreasing methanogenic activity^43^. Hence, the changes in O_2_ availability and moisture contents are important in revealing the processes of CH_4_ production and oxidation in drainage-affected thermokarst lakes and may be key indicators for future CH_4_ modelling. Second, lake drainage can lead to the binding of labile carbon to minerals, reducing substrate availability and thus inhibiting CH_4_ release^44^. This explanation is consistent with that abrupt permafrost thaw resulted in significant increases in iron-bound organic carbon contents by reducing soil moisture on the QTP^45^. Abrupt permafrost thaw reduces soil moisture and improves aeration, allowing oxygen to replace Fe(III) as electron acceptors for microbial respiration and stimulating Fe(II) oxidation to Fe(III), thereby promoting the formation of iron-bound organic carbon^46,47^. Overall, our study suggests that thermokarst lake drainage halves the Q_10_ of CH_4_ release in surface sediments. However, the changes in the response of CH_4_ release to warming during lake drainage are not considered in the simulation of permafrost carbon feedback. Therefore, our study sheds light on the crucial process for understanding the carbon-climate feedback in the changing alpine thermokarst lakes.

### Drivers of CH_4_ release

**Fig. 4:**
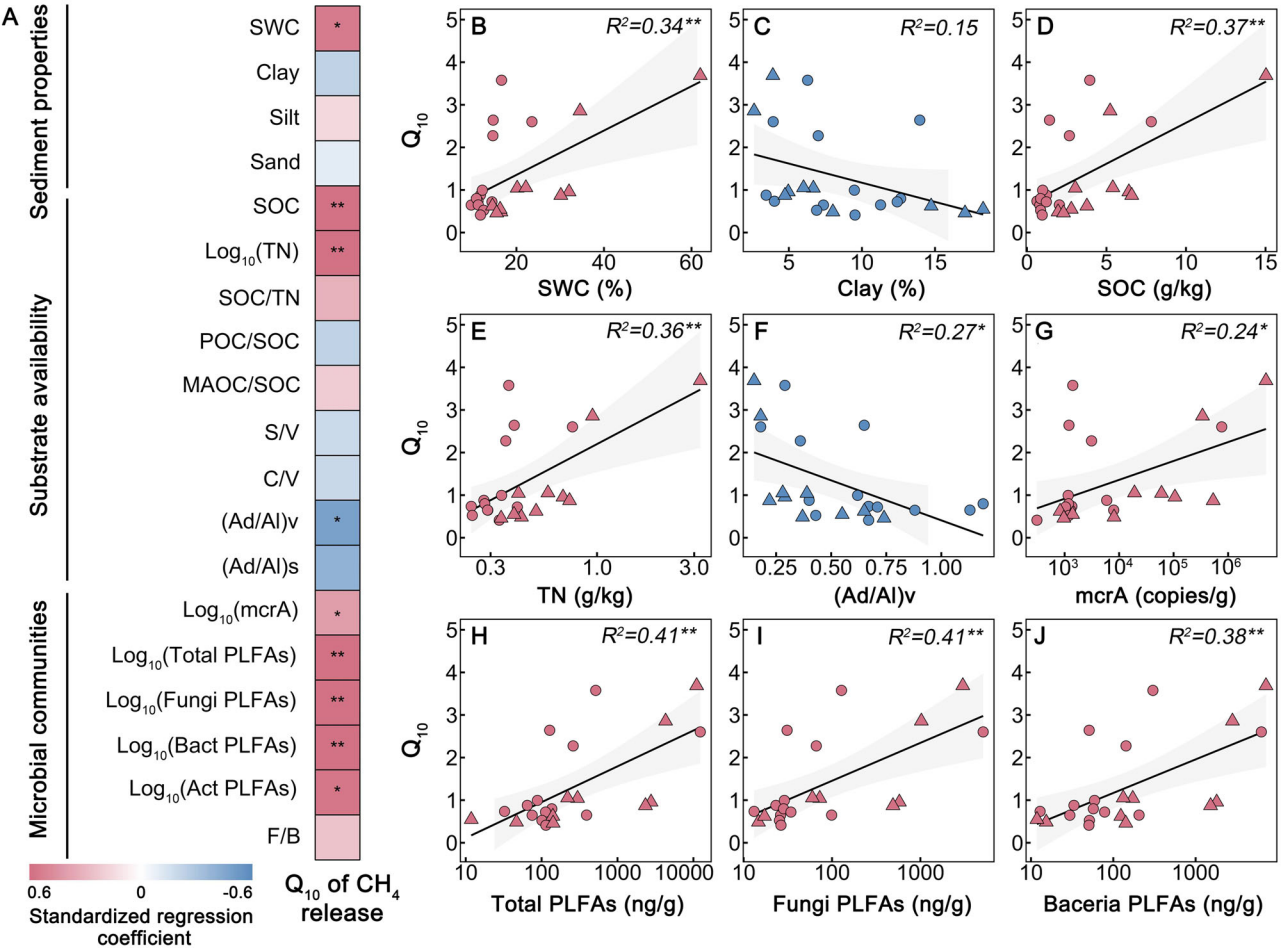
Relationships of temperature sensitivity (Q_10_) with lake sediment properties, substrate availability, and microbial communities. **A** The diagram shows the standardized regression coefficient. **B**–**J** The correlations between Q_10_ and influencing factors. SWC, sediment water content; SOC, soil organic carbon; TN, total nitrogen; POC, particulate organic carbon; MAOC, mineral-associated organic carbon; Bact, bacterial PLFAs; Act, actinomycetes PLFAs; F/B, the ratio of fungal PLFAs to bacterial PLFAs; S/V, the ratio of vanillyls to syringyls in ligninphenol; C/V, the ratio of vanillyls to cinnamyls in ligninphenol; The ratio of acid to aldehyde forms of vanillyls and syringyls [(Ad/Al)v and (Ad/Al)s]. Red and blue indicate positive and negative relationships, respectively. The solid lines and grey area represent the linear regressions and the 95% confidence interval, respectively. Circles and triangles denote BLH-A and BLH-B, respectively. R^2^, the proportion of variance explained. *, ** and *** indicate significant correlation between Q_10_ and the corresponding variable at *p* < 0.05, *p* < 0.01, *p* < 0.001, respectively. Source data are provided as a Source Data file.

**Fig. 5:**
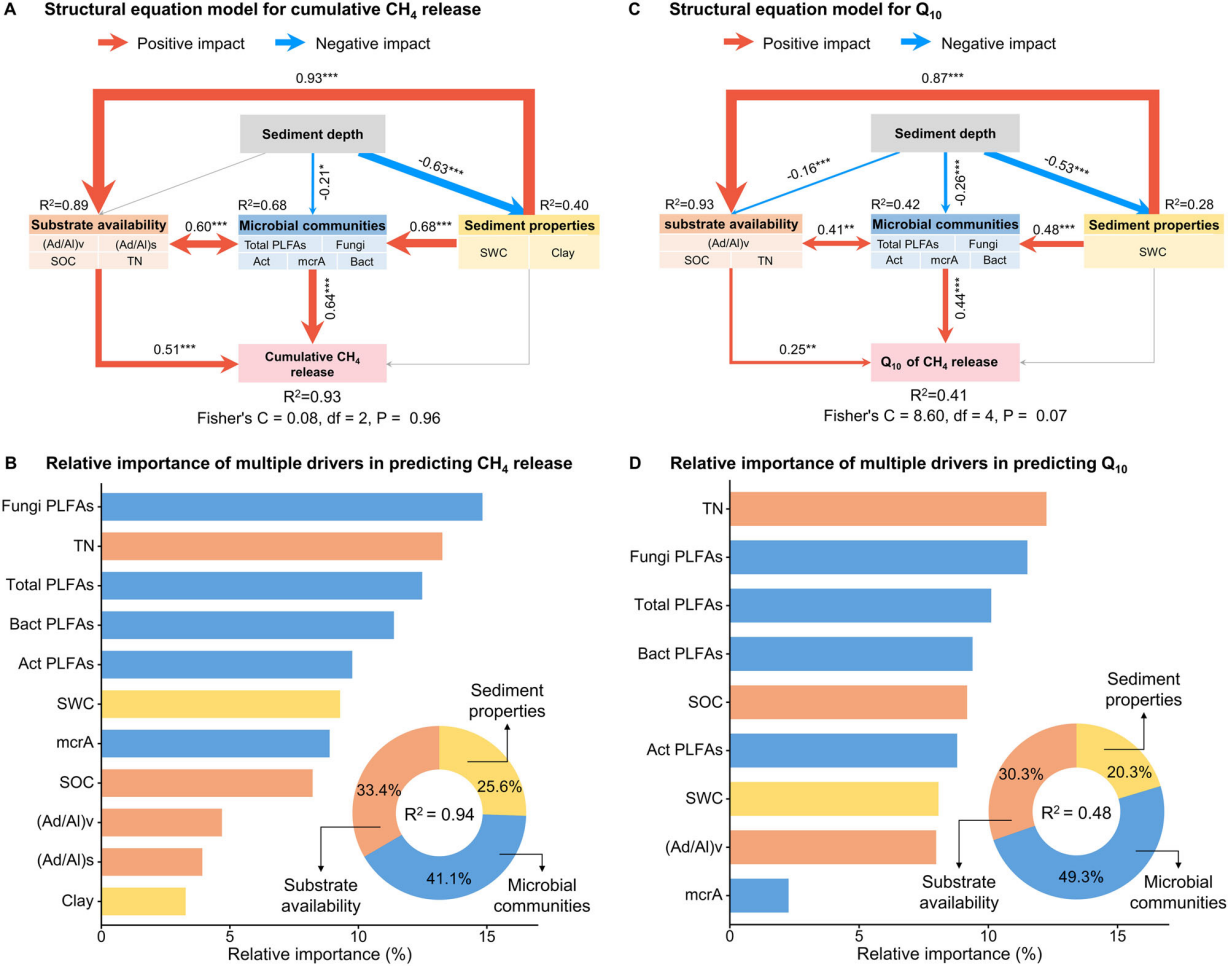
Effects of influencing factors on CH_4_ release and its temperature sensitivity (Q_10_). **A**, **C** Structural equation modelling (SEM) shows the multivariate effects on CH_4_ release and Q_10_. Red and blue lines indicate positive and negative relationships, respectively. Grey lines suggest insignificant paths. The width of the solid line is proportional to the correlation strength. Substrate availability includes the ratio of acid to aldehyde forms of vanillyls and syringyls [(Ad/Al)v], soil organic carbon (SOC), and total nitrogen (TN). Microbial communities are represented by total PLFAs, bacterial PLFAs (Bact), fungal PLFAs (Fungi), actinomycetes PLFAs (Act), and the mcrA gene. Sediment properties include sediment water content (SWC) and clay content (Clay). The goodness-of-fit statistics of the model are displayed below the model. **B**, **D** Relative importance of multiple drivers in predicting CH_4_ release and Q_10_. Variation partitioning modelling evaluates the relative contributions of sediment properties, substrate availability, and microbial communities in explaining variations in CH_4_ release and Q_10_ variation. Source data are provided as a Source Data file.

To reveal the factors controlling the CH_4_ release and Q_10_ in drainage-affected thermokarst lakes, we established the relationships of cumulative CH_4_ release and Q_10_ with potential influencing factors, including sediment properties, substrate availability, and microbial communities (See methods) (Figs. 4, 5, and S7). The results show that the cumulative CH_4_ release is closely related to the sediment properties and substrate availability. Specifically, cumulative CH_4_ release positively correlates with SWC, SOC, and TN (all *p* < 0.001), and negatively correlates with clay content, (Ad/Al)v, and (Ad/Al)s (all *p* < 0.05; Fig. S7). Higher microbial abundance (all *p* < 0.001) and mcrA gene abundance (R^2^ = 0.71, *p* < 0.001) are associated with higher CH_4_ release (Fig. S7). Likewise, substrate availability and microbial communities are also the key predictors of Q_10_ variations (Figs. 4 and 5). Specifically, the Q_10_ of CH_4_ release is positively correlated with microbial abundance, SOC, TN, and SWC (all *p* < 0.01); while is negatively correlated with (Ad/Al)v (R^2^ = 0.27, *p* < 0.05; Fig. 4). To current knowledge, nitrogen plays an important role in CH_4_ production because N-rich compounds are rich in proteins, which can be consumed preferentially by microbes^40^. Thus, our result suggests that TN is a crucial contributor to CH_4_ release from thermokarst lakes. For lake drainage events, sediment moisture is severely influenced due to the changes from a waterlogged environment to terrestrial ecosystems. Low sediment moisture contents caused by lake drainage destroy anaerobic environments that hinder the CH_4_ release^36^. Consistent with this deduction, we find faster CH_4_ release in surface sediment with higher moisture contents (Figs. 1 and 3). Furthermore, we find that clay content is negatively correlated to the CH_4_ release, attributing to that organic carbon is not easily accessible to microbes in clay dominant sediment. Soil organic matter can be stabilized by chemical interaction with clay minerals to form organic-mineral and also physical occlusion within microaggregates^48^. The results suggest that substrate availability, microbial communities, and sediment properties are crucial for controlling CH_4_ release and Q_10_ in drainage-affected thermokarst lakes.

To quantify the determinants and explore the mechanism influencing the CH_4_ release and Q_10_, we conducted an analysis using SEM and variation partitioning modelling. The results show that the three influencing factors together explain 93% of the variances in CH_4_ release (Fig. 5A). Among them, substrate availability (33.4%) and microbial communities (41.1%) have significantly positive effects on CH_4_ release based on the multiple regression models (Fig. 5B). This result is consistent with the CH_4_ release in non-drainage thermokarst lakes on the QTP^24^. Additionally, the SEM explained 41% of the total variation in Q_10_ (Fig. 5C), and substrate availability and microbial communities are responsible for 30.3% and 49.3% of the variances in CH_4_ release, respectively (Fig. 5D). Taken together, results demonstrate that substrate availability and microbial communities are essential factors regulating the changes of CH_4_ release and Q_10_ in the drainage-affected thermokarst lakes. Our study highlights the importance of incorporating microbial communities and substrate availability into Earth System Models when predicting CH_4_ dynamics in thermokarst lakes.

Nonetheless, our study also identifies additional knowledge gaps and perspectives. Firstly, for the drainage-affected and non-drainage thermokarst lakes, future large-scale field surveys are essential for further understanding substrate availability and microbial community composition controlling CH_4_ production and oxidation processes. Secondly, incorporating the CH_4_ release and Q_10_ in thermokarst lakes into models is a priority for predicting future carbon-climate feedback caused by abrupt permafrost thaw. Thirdly, although our study finds that drainage events mitigate CH_4_ release from thermokarst lakes, CO_2_ emissions from the exposed soils could be accelerated. Therefore, comprehensive assessments of greenhouse gases in the changing thermokarst lakes are crucial for a deep understanding of the feedback of permafrost carbon to climate change.

## Methods

### Study region and field drilling

The study region was located in the Beilu River Basin of the central QTP (Fig. 1). There are two major vegetation types in this region, including alpine meadow and alpine wet meadow. The dominant plants are *Kobresia tibetica* and *Carex spp*. This region was covered by continuous permafrost with active layer thickness ranging from 1.5 to 2.5 m and permafrost depth over 20 metres^49^. The mean annual ground temperature (at a depth of 15 m) in the study ranges from −2.0 to −0.5 °C, which is typical of a high-temperature permafrost zone^50^. Permafrost volumetric ice content is more than 30% in a large part of this region^51^, leading to the widespread development of thermokarst lake. From 1969 to 2019, the number and area of thermokarst lakes increased by 159.7% and 121.5% respectively, which was driven by small lakes less than 0.5 ha in size^52^. Thermokarst lakes are characterized by small surface areas and shallow depths, which are susceptible to environmental disturbance. In this study area, the average annual temperature ranged from −6.5 to −4 °C and precipitation from 135 to 470 mm from 1955 to 2019^52^. Due to the influence of the East Asian summer monsoon, precipitation mainly occurs from May to September (Fig. S8), leading to part of thermokarst lakes experiencing large seasonal water level fluctuation annually. Field investigation and remote sensing images revealed that these lakes remain as large water bodies during the July-October, while they are drained in winter. These lakes are referred to be dry and wet cyclically as seasonal drained thermokarst lakes in this study.

Two typical seasonal drained thermokarst lakes of BLH-A and BLH-B were selected (Fig. 1A, B). The lake BLH-A (34.83°N, 92.92°E and 4648 m a.s.l) is located at alpine meadow, with lake basin area of 2530 m^2^. The lake BLH-B (34.83°N, 92.90°E and 4,659 m a.s.l) has a basin area of 552 m^2^ and is situated at alpine wet meadow (Table S1). The distance between the two lakes is approximately 1600 m. The vegetation within lake basins of the two seasonal drained thermokarst lakes is consistent with that around the lake. We collected two sediment cores from the bottom of two seasonal thermokarst lakes by using a drilling rig in May 2021 (Figs. 1,  S9 and *Supplementary Notes*). The cores were drilled with a 10 cm diameter for the uppermost parts and an 8 cm diameter for the lower parts. The sediment core of BLH-A was 387 cm long and the sediment core of BLH-B was 555 cm long. To prevent the introduction of potential contaminants during the drilling, the outer layer of each core was scraped with autoclaved knives. The cores were labelled, packed, and stored in a −20 °C refrigerator until transported back to the laboratory. We divided the sediment cores into four equal parts: one part was subjected to air-drying for subsequent sediment physical and chemical properties measurements, another was preserved at −80 °C for subsequent DNA extraction, and the remaining two were stored at −80 °C as backup.

### Sediment physical and chemical properties

Sediment water content was calculated by measuring the mass of the sediment before and after drying at 105 °C. Sediment texture is determined by chemically removing organic matter (H_2_O_2_ oxidation) and carbonates (HCl dissolution) using a laser particle size metre (Malvern Masterizer 2000, Malvern). Sediment total carbon (TC) and TN content were determined by high-temperature combustion using an elemental analyzer (Vario, Elementar, Hanau, Germany), and SOC content was determined by removing inorganic carbon using dilute hydrochloric acid. We calculated the weight ratio of SOC to TN, which will be referred to as the C/N ratio. The C/N indicates the degree of degradation of organic matter, with C/N decreasing with decomposition. Radiocarbon ages of sediment samples were subjected to accelerator mass spectrometry (AMS) at the Key Laboratory of Western China’s Environmental Systems, Ministry of Education, Lanzhou University. The data were calibrated using the programme CALIB v 7.02 and the IntCal13 curve.

### Sediment substrate availability

We adopted SOC, TN, C/N, POC/SOC, MAOC/SOC, S/V, C/V, (Al/Ad)s, and (Al/Ad)v to characterize sediment substrate availability^53–55^.

#### SOC fractions

To determine the relative contributions of MAOC and POC fraction to total SOC, we fractionated the sediments by size (53 μm) after they were fully dispersed^56^. Briefly, ~6 g air-dried sediment was shaken in 30 ml (5 g/L) sodium hexametaphosphate for 18 h to disperse the sediment completely. The dispersed sediment samples were sieved to 53 μm and rinsed with distilled water. The fraction passing through the sieve (<53 μm) was collected as MAOC, remaining on the sieve was collected as POC. After drying to constant weight in an oven at 60 °C, each fraction was analyzed for organic carbon concentration using an element analyzer (Vario, Elementar, Hanau, Germany) after acid treatment.

#### Lignin phenols analysis

The lignin phenols concentration of sediment samples was quantified by using the copper oxide (CuO) oxidation method^57^. Briefly, about 0.5–1 g freeze-dried sediment was mixed with 1 g of CuO, 0.1 g ammonium iron (II) sulfate hexahydrate, and 15 ml 2 mol/L NaOH in a tetrafluoroethylene reaction kettle. The headspace of the kettle was flushed with N_2_ for 15 min and heated at 170 °C, for 2.5 h. The oxidation products were spiked with 400 μL ethylvanillin as recovery standard, acidified to pH<1 with 6 mol/L hydrochloric acid, and kept in the dark for at least 1 h. After centrifugation, oxidation products were liquid-liquid extracted from the clear supernatant with ethyl acetate three times and concentrated under N_2_ for further analysis. Lignin contents are the sum of the vanillyl (V), syringyl (S), and cinnamyl (C) monomers together^58^. Specifically, the V includes vanillin, acetovanillone, and vanillic acid. The S includes syringaldehyde, acetosyringone, and syringic acid. The C monomer was derived from the sum of p-coumaric acid and ferulic acid. The ratios of acid to aldehyde (Ad/Al) of V and S phenols were used to indicate the degree of lignin degradation and increase with lignin oxidation; The ratios of S/V and C/V were used to indicate the stability of plant substrates^59^.

### Microbial abundance and community composition

We analysed the phospholipid fatty acid (PLFA) in the sediment of thermokarst lakes, which is considered a common method for assessing the microbial abundance and community composition^55,60^. The PLFAs were extracted from sediments using a chloroform-methanol-citrate buffer system following previously described procedures^61^. Before GC analysis, the samples were dissolved in hexane, and calibrated with a standard FAME solution of 19:0. A gas chromatograph and a MIDI Sherlock Microbial Identification System were used for qualitative and quantitative analysis. The PLFAs were classified as bacterial (i13:0, a13:0, i14:0, a15:0, i15:0, 15:1ω6c, i16:0, 16:1ω9c, a17:0, cy17:0, i17:0, 17:1ω6c, i18:0, 18:1ω5c, and 18:1ω7c), fungi (18:1ω9c, 18:2ω6,9c, and 18:3ω6c) and actinomycetes (16:0 Me, 17:0 Me, and 18:0 Me)^62^. Microbial community structure was assessed using the ratios of fungi to bacteria (F/B)^62^.

The functional gene *mcrA* can encode methyl-coenzyme M reductase and is known as a key enzyme in methanogenesis, have been widely employed for quantification of methanogens^63,64^. In this study, to determine the abundance and community composition of methanogenic archaea, we quantified the *mcrA* gene. In detail, sediment DNA was extracted from 3 g of each freeze-dried sediment sample and then purified with a PowerMax Soil DNA Isolation Kit. DNA qualities were evaluated by using a NanoDrop ND-8000 spectrophotometer (Thermo Fisher Scientific, USA). To assess the abundance of methanogenic archaea, the quantitative real-time PCR (qPCR) was performed in a Pharmaceutical Analytics QuantStudio™ 5 Real-Time PCR System (Applied Biosystems, USA). The mcrA genes were amplified by PCR with MLf (5′- GGTGGTGTMGGATTCACACARTAYGCWACAGC -3′) and MLr (5′- TTCATTGCRTAGTTWGGRTAGTT -3′) as primer pairs. For each amplification, purified plasmids containing the target gene were prepared in a 10-fold dilution series for the calibration curve. Based on the calibration curve, the number of gene copies in each sample was calculated.

### Anaerobic sediment incubations

The potential CH_4_ release rates were measured using the method described by Heslop et al^39^. The frozen samples were thawed at 4 °C overnight. From each sample, we prepared four replicates for quality control. About 10 g of fresh sediment was weighed into a 50 ml clamp-mouth anaerobic bottle and added 15 ml of ultrapure water to homogenise the sediment. The bottles were sealed with sterile butyl rubber septa and flushed with pure N_2_ for 20 min. All bottles and butyl rubber stoppers were sterilized at 121 °C for 8 h prior to use and all manipulations were carried out in an anaerobic glove box (N_2_/H_2_, 97/3%) to exclude atmospheric O_2_ contamination. For each sample, the replicate samples were incubated anaerobically at 5 °C, 15 °C, and 25 °C in the dark simultaneously. The incubation temperature represents the mean annual temperature, maximum summer temperature, and warming conditions at the bottom of the thermokarst lake on the QTP, respectively^65^. To measure CH_4_ concentration, we sampled 1 mL headspace gas with a syringe and injected it into the gas chromatograph (GC, Agilent-7890A). Subsequently, 1 mL of N_2_ was added to the bottles to maintain air pressure equilibrium in the bottle. We measured the changes in the CH_4_ production at nine time points during a 150-day incubation. The difference in headspace CH_4_ concentration between two sampling time points during the incubation period was used to calculate the CH_4_ release rate. The calculated formula is as follows^66^:

1 $$F=\frac{{{{\rm{dc}}}}}{dt}\times \frac{Vh}{Ws}\times \frac{MW}{MV}\times \frac{Tst}{Tst+T}$$

where *F* is the potential CH_4_ release rate in μg CH_4_ g^−1^ dry sediment d^−1^, *dc/dt* is the change in CH_4_ concentration in the headspace of the anaerobic bottles with incubation time. The *Vh* is headspace volume, *Ws* is sediment wet weight, and *MW* and *MV* are the molar mass and molar volume of CH_4_ at standard conditions respectively. *Tst* is the standard temperature (273 K), and *T* is the incubation temperature (°C).

The Q_10_ of CH_4_ release for laboratory incubation was calculated based on the potential CH_4_ release rate at the two incubation temperatures as follows^67^:

2 $${Q}_{10}={\left(\frac{{R}_{W}}{{R}_{C}}\right)}^{[10/{T}_{W}-{T}_{C}]}$$

where *R*_*W*_ and *R*_*C*_ are the average CH_4_ release rate (μg CH_4_ g^−1^ dry sediment d^−1^) at warmer (*T*_*W*_) and cold (*T*_*C*_) temperatures (°C), respectively.

### Data synthesis of Q_10_ in thermokarst lakes

To show the effect of drainage on Q_10_ of CH_4_ release in thermokarst lake sediments, we synthesized the published Q_10_ data of sediment CH_4_ release in the non-drainage thermokarst lakes. Given large variations in CH_4_ release from thermokarst lakes with different vegetation type^24^, we only compiled the Q_10_ data on CH_4_ release from lake sediments located in alpine meadow and wet meadow regions on the QTP. There is limited study reporting the Q_10_ of CH_4_ release of thermokarst lake sediments^23^. We compiled our unpublish Q_10_ data of non-drainage thermokarst lakes on the QTP based on similar laboratory incubation (Table S2). Considering the vertical variation of Q_10_ values in this study, the surface 0-30 cm data was used for the comparison.

### Statistical analyses

All statistical analyses were performed using the software R 4.1.3^68^. Data were presented as mean ± SE and all statistical tests’s significance was determined at the α = 0.05 level. Before analysis, the Quantile-Quantile Plot^69^ and Levene’s tests (function leveneTest)^70^ were used to check the normality and homogeneity of variance for all variables, respectively. Wilcox test was conducted to examine the significance of the difference in sediment properties, substrate availability, and microbial communities with different depths using the function of *wilcox.test* in R package “stats”^68^. A paired t-test was conducted to examine the significance of the difference in Q_10_ values with different depths and lake types using the function of *t.test* in the R package “rstatix”^71^. General linear models were conducted to examine the correlations of cumulative CH_4_ release and Q_10_ values with sediment properties, substrate availability, and microbial communities using the function “lm”^68^. We examined the normality and homoscedasticity of the residuals of all the linear models, and the data were logarithmically transformed when necessary. Correlations between two variables were assessed using “Pearson” correlation analysis. We also established the age-depth model for both thermokarst lake sediments using the function “lm” and “nls” in R package “stats”^68^ (Fig. S10) and used the models to calculate carbon ages for other sediment depths.

To further quantify the relative contributions of sediment properties, substrate availability, and microbial communities to cumulative CH_4_ release and Q_10_, we combined a piecewise SEM, multiple linear regression (MLR), and hierarchical partitioning to assess their relationships and relative contribution. In the initial conceptual model, we hypothesized that the microbial communities (mcrA, total PLFAs, bacterial PLFAs, fungal PLFAs, and actinomycetes PLFAs), substrate availability (SOC, TN, (Ad/Al)v and (Ad/Al)s) and sediment properties (sediment moisture and clay content) had direct effects on CH_4_ release and Q_10_. The goodness of fit of the model was evaluated using Fisher’s C-statistic and the whole-model *P* value. The SEM, MLR, and hierarchical partitioning were performed using the R packages “piecewiseSEM”^72^, “stats”^68^, “relaimpo”^73^, and “rdacca.hp”^74^, respectively.

## Supplementary information

Supplementary Information

Peer Review file

## Data Availability

All data supporting the findings are available online in Supplementary information and the Figshare data repository (10.6084/m9.figshare.26880526)^75^. Source data are provided in this paper.

## Code availability

Data analysis was performed using R 4.1.3. which is publicly available at https://www.r-project.org. The supporting code is provided at Figshare (10.6084/m9.figshare.26892715)^76^.

## References

[1] Obu, J. et al. Northern Hemisphere permafrost map based on TTOP modelling for 2000–2016 at 1km^2^ scale. *Earth Sci. Rev.* **193**, 299–316 (2019).

[2] Mishra, U. et al. Spatial heterogeneity and environmental predictors of permafrost region soil organic carbon stocks. *Sci. Adv.* **7**, eaaz5236 (2021). 33627437 10.1126/sciadv.aaz5236 PMC7904252

[3] Jones, B. M. et al. Lake and drained lake basin systems in lowland permafrost regions. *Nat. Rev. Earth Environ.* **3**, 85–98 (2022).

[4] Webb, E. E. & Liljedahl, A. K. Diminishing lake area across the northern permafrost zone. *Nat. Geosci.* **16**, 202–209 (2023).

[5] Pekel, J. F., Cottam, A., Gorelick, N. & Belward, A. S. High-resolution mapping of global surface water and its long-term changes. *Nature* **540**, 418–422 (2016). 27926733 10.1038/nature20584

[6] Olefeldt, D. et al. Circumpolar distribution and carbon storage of thermokarst landscapes. *Nat. Commun.* **7**, 13043 (2016). 27725633 10.1038/ncomms13043 PMC5062615

[7] Walter Anthony, K. et al. Methane emissions proportional to permafrost carbon thawed in Arctic lakes since the 1950s. *Nat. Geosci.* **9**, 679–682 (2016).

[8] Walter Anthony, K. M. et al. Decadal-scale hotspot methane ebullition within lakes following abrupt permafrost thaw. *Environ. Res. Lett.* **16**, 035010 (2021).

[9] Yang, G. et al. Characteristics of methane emissions from alpine thermokarst lakes on the Tibetan Plateau. *Nat. Commun.* **14**, 3121 (2023). 37253726 10.1038/s41467-023-38907-6 PMC10229571

[10] Brosius, L. S. et al. Panarctic lakes exerted a small positive feedback on early Holocene warming due to deglacial release of methane. *Commun. Earth Environ.* **4**, 271 (2023).

[11] Walter, K. M. et al. Thermokarst lakes as a source of atmospheric CH_4_ during the last deglaciation. *Science* **318**, 633–636 (2007). 17962561 10.1126/science.1142924

[12] Wik, M., Varner, R. K., Anthony, K. W., MacIntyre, S. & Bastviken, D. Climate-sensitive northern lakes and ponds are critical components of methane release. *Nat. Geosci.* **9**, 99–105 (2016).

[13] Turetsky, M. R. et al. Carbon release through abrupt permafrost thaw. *Nat. Geosci.* **13**, 138–143 (2020).

[14] Chen, Y., Cheng, X., Liu, A., Chen, Q. & Wang, C. Tracking lake drainage events and drained lake basin vegetation dynamics across the Arctic. *Nat. Commun.* **14**, 7359 (2023). 37968270 10.1038/s41467-023-43207-0 PMC10652023

[15] Fuchs, M. et al. Organic carbon and nitrogen stocks along a Thermokarst Lake sequence in Arctic Alaska. *J. Geophys. Res. Biogeosci.* **124**, 1230–1247 (2019). 31341754 10.1029/2018JG004591 PMC6618060

[16] Skeeter, J., Christen, A., Laforce, A.-A., Humphreys, E. & Henry, G. Vegetation influence and environmental controls on greenhouse gas fluxes from a drained thermokarst lake in the western Canadian Arctic. *Biogeosciences* **17**, 4421–4441 (2020).

[17] Loiko, S., Klimova, N., Kuzmina, D. & Pokrovsky, O. Lake drainage in permafrost regions produces variable plant communities of high biomass and productivity. *Plants* **9**, 867 (2020). 32650600 10.3390/plants9070867 PMC7411715

[18] Chen, Y., Liu, A. & Cheng, X. Vegetation grows more luxuriantly in Arctic permafrost-drained lake basins. *Glob. Chang. Biol.* **27**, 5865–5876 (2021). 34411382 10.1111/gcb.15853 PMC9291482

[19] Kao-Kniffin, J. et al. Archaeal and bacterial communities across a chronosequence of drained lake basins in arctic Alaska. *Sci. Rep.* **5**, 18165 (2015). 26681584 10.1038/srep18165 PMC4683534

[20] Patil, V. P. et al. The effect of drying Boreal lakes on plants, soils, and microbial communities in lake margin habitats. *J. Geophys Res Biogeosci.* **129**, e2023JG007819 (2024).

[21] Patzner, M. S. et al. Iron mineral dissolution releases iron and associated organic carbon during permafrost thaw. *Nat. Commun.* **11**, 6329 (2020). 33303752 10.1038/s41467-020-20102-6 PMC7729879

[22] Yang, S. et al. Microbial methane cycling in sediments of Arctic thermokarst lagoons. *Glob. Chang Biol.* **24**, 2714–2731 (2023). 10.1111/gcb.16649 36811358

[23] Xu, Q. et al. Temperature sensitivity of methanogenesis and anaerobic methane oxidation in thermokarst lakes modulated by surrounding vegetation on the Qinghai-Tibet Plateau. *Sci. Total Environ.* **907**, 167962 (2024). 10.1016/j.scitotenv.2023.167962 39491188

[24] Mu, C. et al. High carbon emissions from thermokarst lakes and their determinants in the Tibet Plateau. *Glob. Chang Biol.* **29**, 2732–2745 (2023). 36854541 10.1111/gcb.16658

[25] Davidson, E. A. & Janssens, I. A. Temperature sensitivity of soil carbon decomposition and feedbacks to climate change. *Nature* **440**, 165–173 (2006). 16525463 10.1038/nature04514

[26] Xu, X. et al. Reviews and syntheses: Four decades of modeling methane cycling in terrestrial ecosystems. *Biogeosciences* **13**, 3735–3755 (2016).

[27] Niu, S. et al. Temperature responses of ecosystem respiration. *Nat. Rev. Earth Environ.* **5**, 559–571 (2024).

[28] Jones, C. D., Cox, P. & Huntingford, C. Uncertainty in climate-carbon-cycle projections associated with the sensitivity of soil respiration to temperature. *Tellus B* **55**, 642–648 (2003).

[29] Schädel, C. et al. Earth system models must include permafrost carbon processes. *Nat. Clim. Change* **14**, 114–116 (2024).

[30] Yan, Y., You, Q., Wu, F., Pepin, N. & Kang, S. Surface mean temperature from the observational stations and multiple reanalyses over the Tibetan Plateau. *Clim. Dyn.* **55**, 2405–2419 (2020).

[31] Mu, C. et al. The status and stability of permafrost carbon on the Tibetan Plateau. *Earth Sci. Rev.* **211**, 103433 (2020).

[32] Wei, Z. et al. Sentinel‐based Inventory of thermokarst lakes and ponds across permafrost landscapes on the Qinghai‐Tibet Plateau. *Earth Space Sci.* **8**, e2021EA001950 (2021).

[33] Mu, M. et al. Thermokarst lake changes along the Qinghai-Tibet Highway during 1991–2020. *Geomorphology* **441**, 108895 (2023).

[34] Șerban, R. D., Jin, H., Șerban, M. & Luo, D. Shrinking thermokarst lakes and ponds on the northeastern Qinghai‐Tibet plateau over the past three decades. *Permafr. Periglac.* **32**, 601–617 (2021).

[35] Mu, C. et al. Carbon and nitrogen properties of permafrost over the Eboling Mountain in the upper reach of Heihe River Basin, Northwestern China. *Arct. Antarct. Alp. Res* **47**, 203–211 (2015).

[36] Keuschnig, C. et al. Reduced methane emissions in former permafrost soils driven by vegetation and microbial changes following drainage. *Glob. Chang Biol.* **28**, 3411–3425 (2022). 35285570 10.1111/gcb.16137 PMC9314937

[37] Evans, P. N. et al. An evolving view of methane metabolism in the Archaea. *Nat. Rev. Microbiol* **17**, 219–232 (2019). 30664670 10.1038/s41579-018-0136-7

[38] Conrad, R. Importance of hydrogenotrophic, aceticlastic and methylotrophic methanogenesis for methane production in terrestrial, aquatic and other anoxic environments: A mini review. *Pedosphere* **30**, 25–39 (2020).

[39] Heslop, J. K. et al. Thermokarst lake methanogenesis along a complete talik profile. *Biogeosciences* **12**, 4317–4331 (2015).

[40] Moras, S., Zellmer, U. R., Hiltunen, E., Grasset, C. & Sobek, S. Predicting methane formation rates of freshwater sediments in different biogeographic regions. *J. Geophys. Res. Biogeosci* **129**, e2023JG007463 (2024).

[41] Isidorova, A., Grasset, C., Mendonça, R. & Sobek, S. Methane formation in tropical reservoirs predicted from sediment age and nitrogen. *Sci. Rep.* **9**, 11017 (2019). 31358820 10.1038/s41598-019-47346-7 PMC6662704

[42] Chen, H., Xu, X., Fang, C., Li, B. & Nie, M. Differences in the temperature dependence of wetland CO2 and CH4 emissions vary with water table depth. *Nat. Clim. Change* **11**, 766–771 (2021).

[43] Liu, C.-T., Miyaki, T., Aono, T. & Oyaizu, H. Evaluation of methanogenic strains and their ability to endure aeration and water stress. *Curr. Microbiol*. **56**, 214–218 (2008). 17990030 10.1007/s00284-007-9059-7

[44] Opfergelt, S. The next generation of climate model should account for the evolution of mineral-organic interactions with permafrost thaw. *Environ. Res Lett.* **15**, 091003 (2020).

[45] Liu, F. et al. Divergent changes in particulate and mineral-associated organic carbon upon permafrost thaw. *Nat. Commun.* **13**, 5073 (2022). 36038568 10.1038/s41467-022-32681-7 PMC9424277

[46] Dong, H. et al. Coupled iron cycling and organic matter transformation across redox interfaces. *Nat. Rev. Earth Environ.* **4**, 659–673 (2023).

[47] Patzner, M. S. et al. Seasonal fluctuations in iron cycling in thawing permafrost peatlands. *Environ. Sci. Technol.* **56**, 4620–4631 (2022). 35290040 10.1021/acs.est.1c06937 PMC9097474

[48] Singh, M. et al. Stabilization of soil organic carbon as influenced by clay mineralogy. *Adv. Agron.* **148**, 33–84 (2018).

[49] Zhao, L. et al. A synthesis dataset of permafrost thermal state for the Qinghai–Tibet (Xizang) Plateau, China. *Earth Syst. Sci. Data* **13**, 4207–4218 (2021).

[50] Ran, Y. et al. New high-resolution estimates of the permafrost thermal state and hydrothermal conditions over the Northern Hemisphere. *Earth Syst. Sci. Data* **14**, 865–884 (2022).

[51] Lu, J. H., Cheng, H., Niu, F. J., Lin, Z. J. & Liu, H. Zoning evaluation on occurrence degree of thermokarst lake along Qinghai Tibet Railway. *J. Catastrophol.* **27**, 60–64 (2012).

[52] Luo, J. et al. Abrupt increase in thermokarst lakes on the central Tibetan Plateau over the last 50 years. *Catena* **217**, 106497 (2022).

[53] Chen, L. et al. Determinants of carbon release from the active layer and permafrost deposits on the Tibetan Plateau. *Nat. Commun.* **7**, 13046 (2016). 27703168 10.1038/ncomms13046 PMC5059472

[54] Dai, G. et al. Plant-derived lipids play a crucial role in forest soil carbon accumulation. *Soil Biol. Biochem* **168**, 108645 (2022).

[55] Zhang, Q. et al. Whole- soil- profile warming does not change microbial carbon use efficiency in surface and deep soils. *Proc. Natl Acad. Sci.* **120**, e2302190120 (2023). 37523548 10.1073/pnas.2302190120 PMC10410710

[56] Poeplau, C. et al. Isolating organic carbon fractions with varying turnover rates in temperate agricultural soils – A comprehensive method comparison. *Soil Biol. Biochem* **125**, 10–26 (2018).

[57] Feng, X. et al. Source to sink: Evolution of lignin composition in the Madre de Dios River system with connection to the Amazon basin and offshore. *J. Geophys Res Biogeosci.* **121**, 1316–1338 (2016).

[58] Feng, X. & Simpson, M. J. The distribution and degradation of biomarkers in Alberta grassland soil profiles. *Org. Geochem* **38**, 1558–1570 (2007).

[59] Ma, T. et al. Phosphorus supply suppressed microbial necromass but stimulated plant lignin phenols accumulation in soils of alpine grassland on the Tibetan Plateau. *Geoderma* **431**, 116376 (2023).

[60] Qin, S. et al. Temperature sensitivity of permafrost carbon release mediated by mineral and microbial properties. *Sci. Adv.* **7**, eabe3596 (2021). 34362729 10.1126/sciadv.abe3596 PMC8346221

[61] Frostegård, A. & Bååth, E. The use of phospholipid fatty acid analysis to estimate bacterial and fungal biomass in soil. *Biol. Fertil.* **22**, 59–65 (1996).

[62] Willers, C., Jansen van Rensburg, P. J. & Claassens, S. Phospholipid fatty acid profiling of microbial communities–a review of interpretations and recent applications. *J. Appl Microbiol* **119**, 1207–1218 (2015). 26184497 10.1111/jam.12902

[63] Zhu, Y. et al. Disproportionate increase in freshwater methane emissions induced by experimental warming. *Nat. Clim. Change* **10**, 685–690 (2020).

[64] Obregon, D. et al. Functionality of methane cycling microbiome during methane flux hot moments from riparian buffer systems. *Sci. Total Environ.* **870**, 161921 (2023). 36739023 10.1016/j.scitotenv.2023.161921

[65] Lin, Z. J., Niu, F. J., Fang, J. H., Luo, J. & Yin, G. A. Interannual variations in the hydrothermal regime around a thermokarst lake in Beiluhe, Qinghai-Tibet Plateau. *Geomorphology* **276**, 16–26 (2017).

[66] Zhang, H., Yao, X., Zeng, W., Fang, Y. & Wang, W. Depth dependence of temperature sensitivity of soil carbon dioxide, nitrous oxide and methane emissions. *Soil Biol. Biochem* **149**, 107956 (2020).

[67] Duc, N. T., Crill, P. & Bastviken, D. Implications of temperature and sediment characteristics on methane formation and oxidation in lake sediments. *Biogeochemistry* **100**, 185–196 (2010).

[68] Team RC. A Language and Environment for Statistical Computing. R Foundation for Statistical Computing, Vienna, Austria. https://www.R-project.org/ (2024).

[69] Wickham H. ggplot2: Elegant Graphics for Data Analysis. Springer-Verlag New York. (2016).

[70] Fox, J. W. S. An R Companion to Applied Regression, Third edition. Sage, Thousand Oaks CA. https://socialsciences.mcmaster.ca/jfox/Books/Companion/ (2019).

[71] Kassambara A. Pipe-Friendly Framework for Basic Statistical Tests. R package version 0.7.2. (2023).

[72] Lefcheck, J. S. & Freckleton, R. piecewiseSEM: Piecewise structural equation modelling in r for ecology, evolution, and systematics. *Methods Ecol. Evol.* **7**, 573–579 (2015).

[73] Ulrike, G. Relative importance for linear regression in R: The Package relaimpo. *J. Stat. Softw.* **17**, 1–27 (2006).

[74] Lai, J., Zou, Y., Zhang, J. & Peres‐Neto, P. R. Generalizing hierarchical and variation partitioning in multiple regression and canonical analyses using the rdacca.hp R package. *Methods Ecol. Evol.* **13**, 782–788 (2022).

[75] Mu, M. et al. Thermokarst lake drainage halves the temperature sensitivity of CH_4_ release on the Qinghai-Tibet Plateau. *Figshare*. 10.6084/m9.figshare.26880526 (2024).10.1038/s41467-025-57356-x40011466

[76] Mu, M. et al. Thermokarst lake drainage halves the temperature sensitivity of CH_4_ release on the Qinghai-Tibet Plateau. Code. *Figshare*. 10.6084/m9.figshare.26892715 (2024).10.1038/s41467-025-57356-x40011466

