# Supplementary material for: Thermokarst lake drainage halves the temperature sensitivity of CH4 release on the Qinghai-Tibet Plateau
Source: Nat Commun. 2025 Feb 26;16:1992. doi: 10.1038/s41467-025-57356-x (PMC11865292; doi:10.1038/s41467-025-57356-x)
Supplement: Supplementary file 1 — Supplementary Information [file 41467_2025_57356_MOESM1_ESM.pdf]

Supplementary Information for

Thermokarst lake drainage halves the temperature sensitivity of CH_4 release on the Qinghai-Tibet Plateau

Mei Mu[1], Cuicui Mu[1,2,3*], Hebin Liu[1], Pengsi Lei[1], Yongqi Ge[1], Zhensong Zhou[1], Xiaoqing Peng[1], Tian Ma[4]

[1] Key Laboratory of Western China's Environmental Systems (Ministry of Education), College of Earth and Environmental Sciences, Observation and research station on Eco-Environment of Frozen Ground in the Qilian Mountains, Lanzhou University, Lanzhou, 730000, China
[2] State Key Laboratory of Cryospheric Science, Northwest Institute of Eco-Environment and Resources, Chinese Academy of Sciences, Lanzhou, 730000, China
[3] Academy of Plateau Science and Sustainability, Qinghai Normal University, Xining, China, 810016, China
[4] State Key Laboratory of Herbage Improvement and Grassland Agroecosystems, College of Pastoral Agriculture Science and Technology, Lanzhou University, Lanzhou 730020, China

*Corresponding author. Email: mucc@lzu.edu.cn.

This PDF file includes:

Supplementary Notes
Figures S1-S10
Table S1 and S2

Supplementary Notes

Drilling methods: We used an exploration drill rig (Wendeng GJ-240, China) with a single core barrel to sample the sediment cores of thermokarst lake. The rig is self-propelled by tracks and has a weight of around 750 kg. The hydraulic rig, powered by a diesel engine, and uses rotary core drilling methods. The rig has a capability of 420 m with a wireline system.

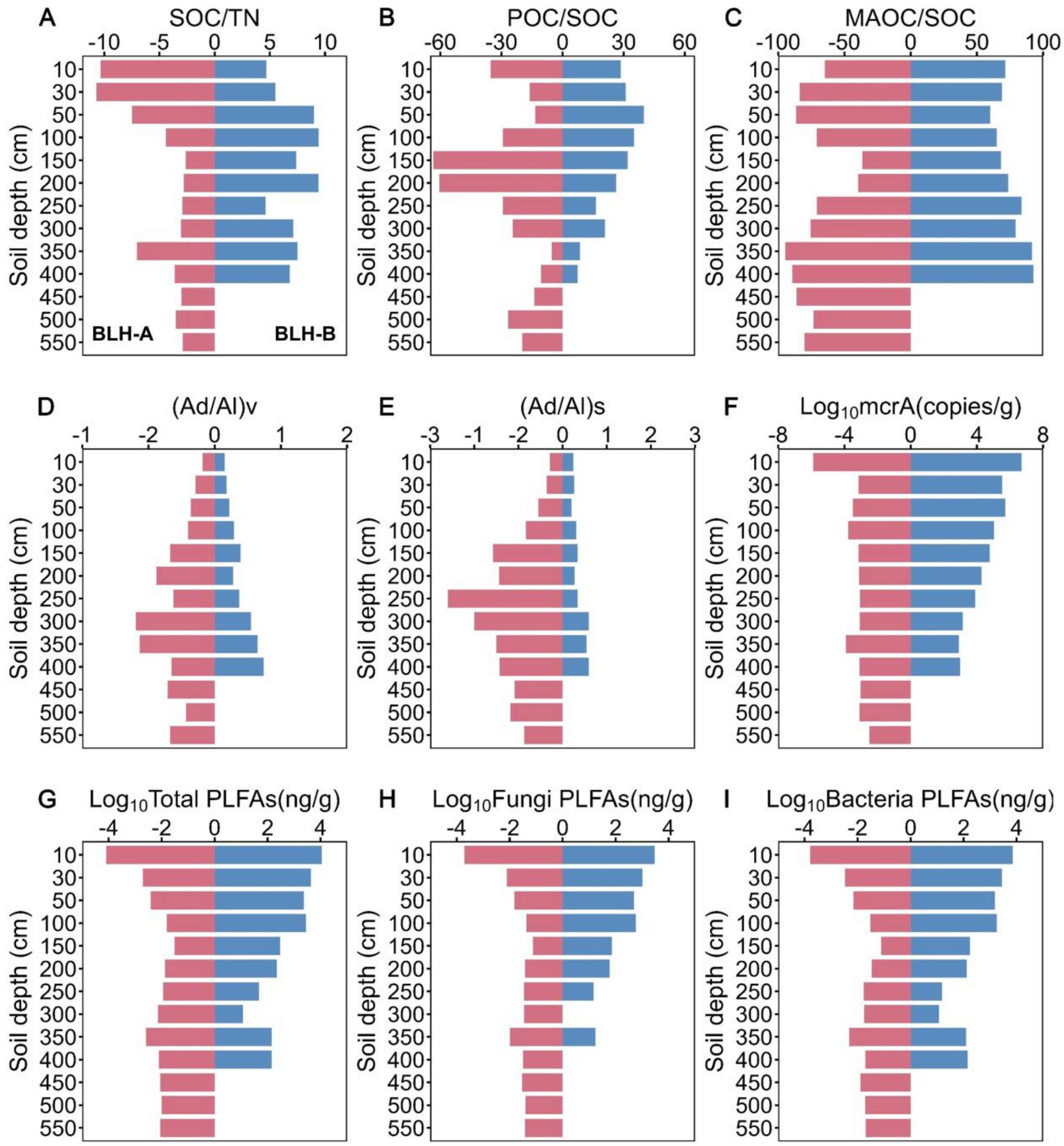

Fig. S1.

Sediment substrate availability and microbial communities in thermokarst lake cores with different depths. **(A)** SOC/TN, the ratio of sediment organic carbon to total nitrogen; **(B)** POC/SOC, proportion of particulate organic carbon to sediment organic carbon; **(C)** MAOC/SOC, proportion of mineral-associated organic carbon to sediment organic carbon; **(D)** (Ad/Al)v, the ratio of acid to aldehyde forms of vanillyls; **(E)** (Ad/Al)s, the ratio of acid to aldehyde forms of syringyls; **(F)** Log_{10}mcrA, ; **(G–I)** Microbial abundance of total, fungi, and bacteria. Red and blue indicate BLH-A and BLH-B lake, respectively. Source data are provided as a Source Data file.

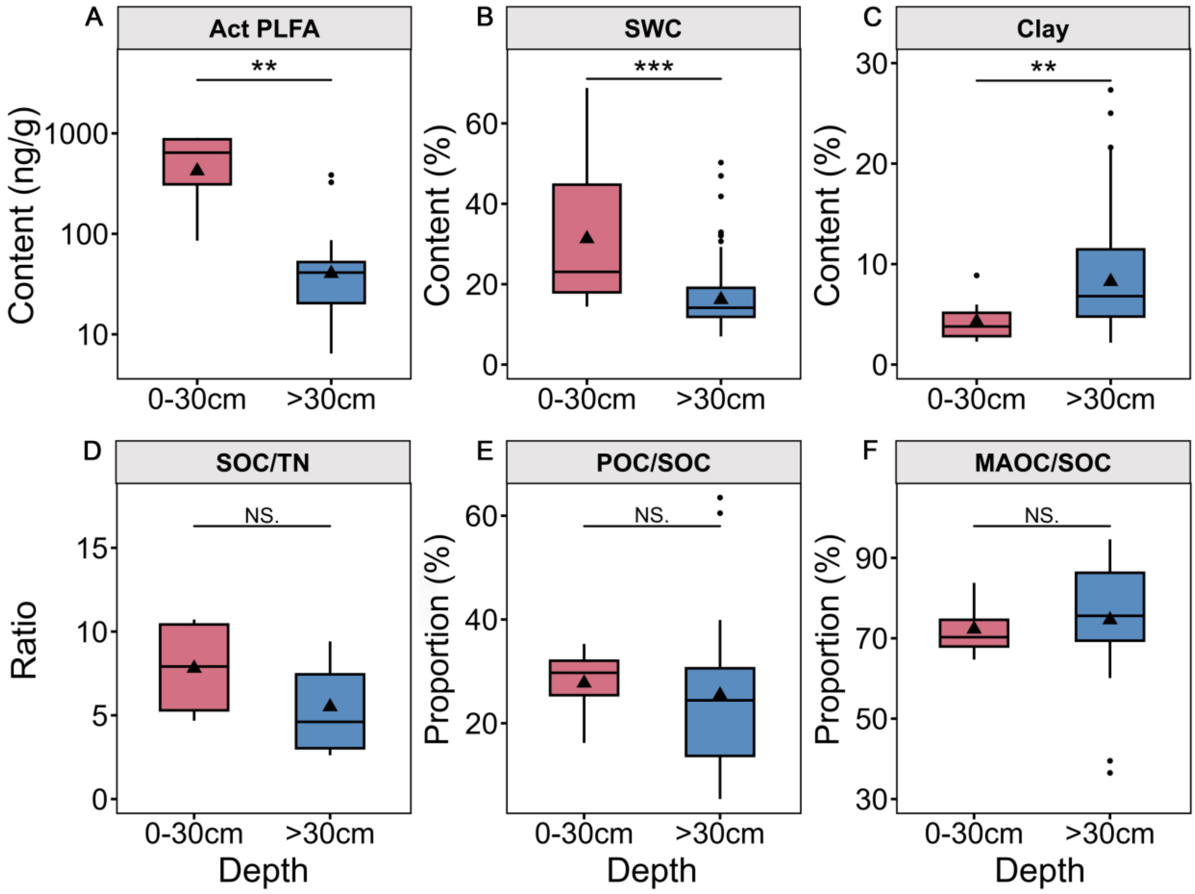

Fig. S2.

Sediment properties, substrate availability, and microbial communities in surface sediments (0–30 cm) and deeper sediments (>30 cm) from the drainage-affected thermokarst lake. **(A)** Act PLFAs, actinomycetes PLFAs; **(B)** SWC, sediment water content; **(C)** Clay, clay content. **(D)** SOC/TN, the ratio of sediment organic carbon to total nitrogen; **(E)** POC/SOC, proportion of particulate organic carbon to sediment organic carbon; **(F)** MAOC/SOC, proportion of mineral-associated organic carbon to sediment organic carbon. The solid line and triangle in the box represent the median and mean of each dataset, respectively. Box represents the interquartile range, with red and blue indicating surface 0-30 cm layer and deeper >30 cm layer, respectively. The upper and lower whisker caps indicate the 1.5 interquartile range of upper and lower quartile, respectively. Dots outside whiskers indicate outliers. *p<0.05, **p<0.01, ***p<0.001. Source data are provided as a Source Data file.

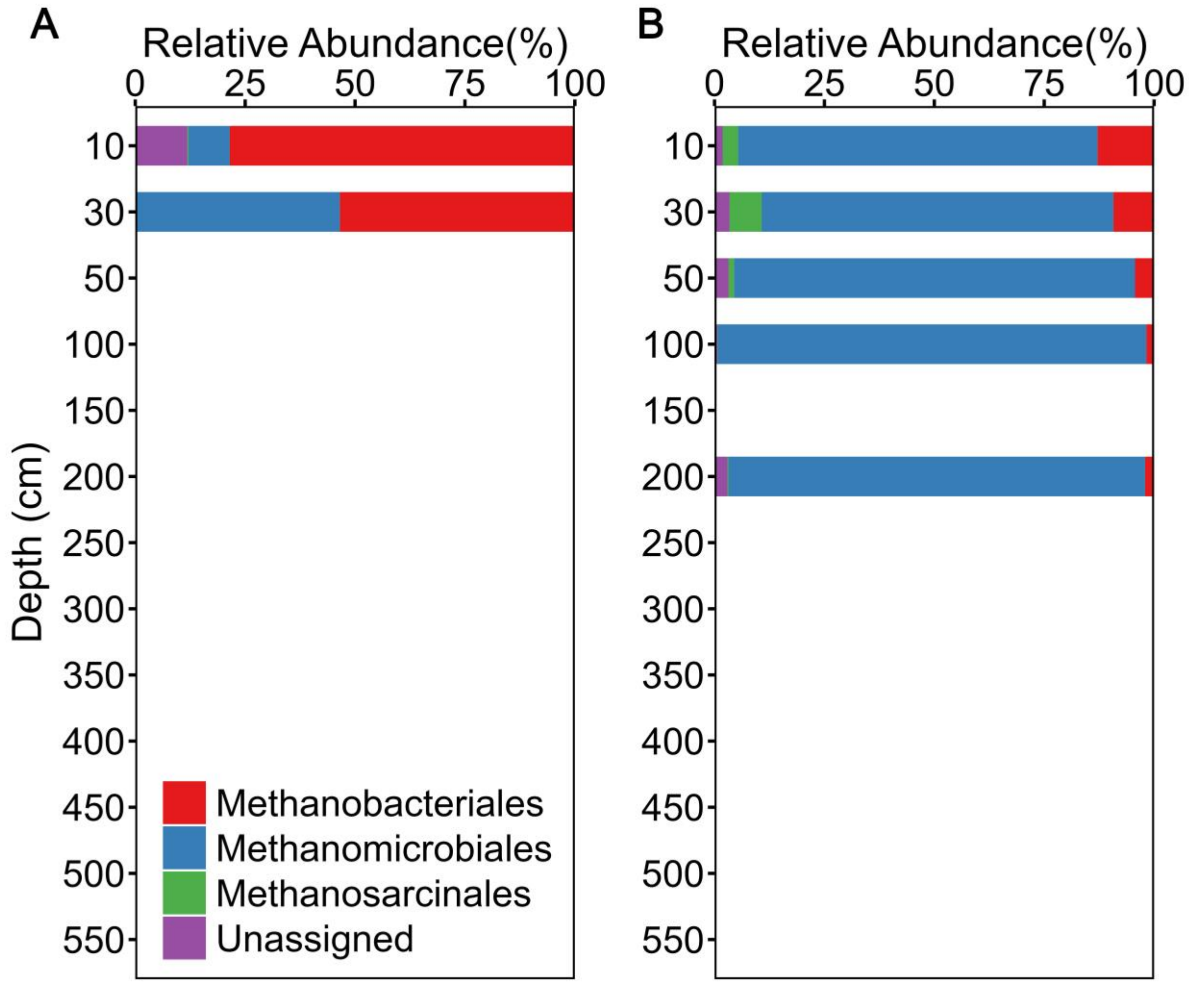

Fig. S3.

The abundance of the methanogens community composition at different depths from thermokarst lake BLH-A and BLH-B. (A) Methanogens community composition at depths of 10 cm and 30 cm in BLH-A; **(B)** Methanogens community composition at depths of 10, 30, 50, 100, and 200 cm in BLH-B. Source data are provided as a Source Data file.

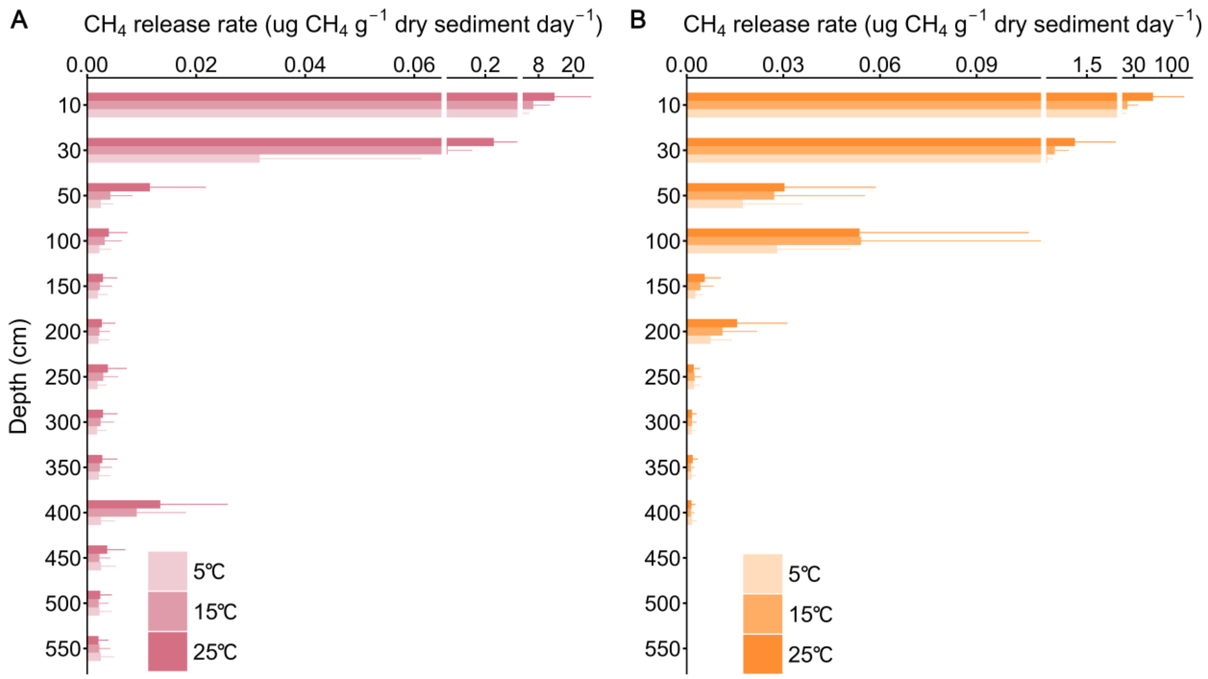

Fig. S4.

CH_4 release rates obtained from anaerobic incubations. (A-B) Potential CH_4 release rates from different sediment depths of thermokarst lakes BLH-A and BLH-B. The different colors represent the CH_4 release rates at the incubation temperatures of 5, 15, and 25 °C. Values are means ± standard errors (SE) (n=4). Source data are provided as a Source Data file.

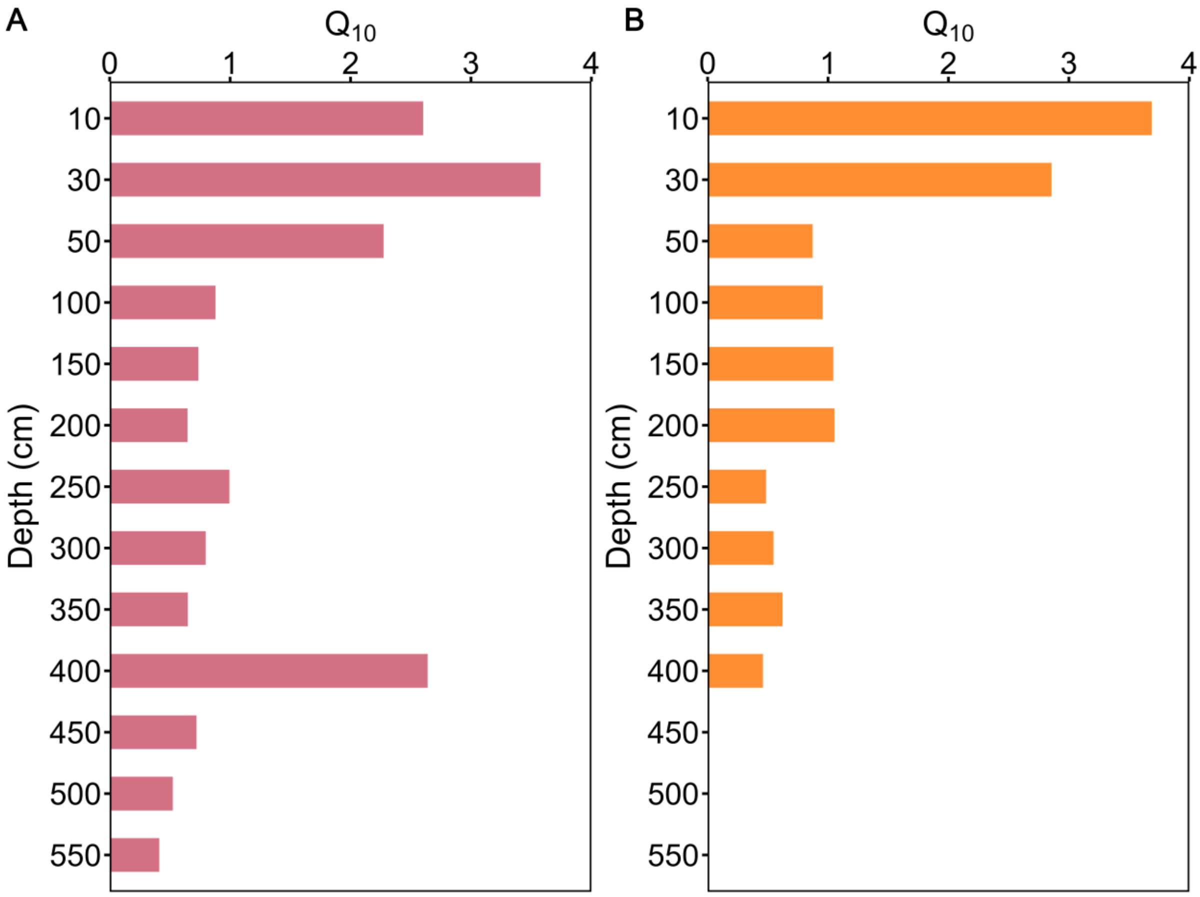

Fig. S5.

Temperature sensitivity (Q_{10}) of CH_4 release from different sediment depths of thermokarst lakes BLH-A and BLH-B. (A) BLH-A; **(B)** BLH-B. Source data are provided as a Source Data file.

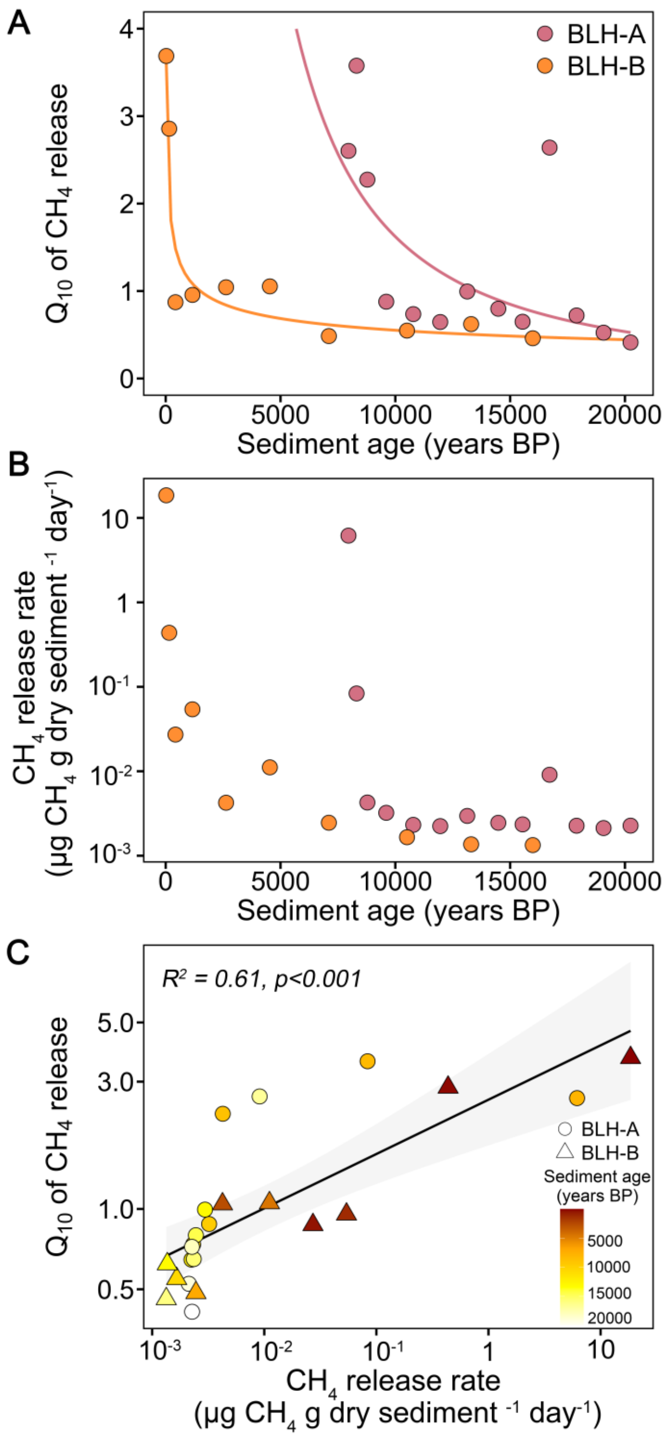

Fig. S6.

Relationships between the CH_4 release rate and its temperature sensitivity (Q_{10}) and sediment age. (A) Relationship between the Q_{10} of CH_4 release and sediment age; **(B)** Relationship between the CH_4 release rate and sediment age; **(C)** Changes in Q_{10} and CH_4 release rates with sediment age. Source data are provided as a Source Data file.

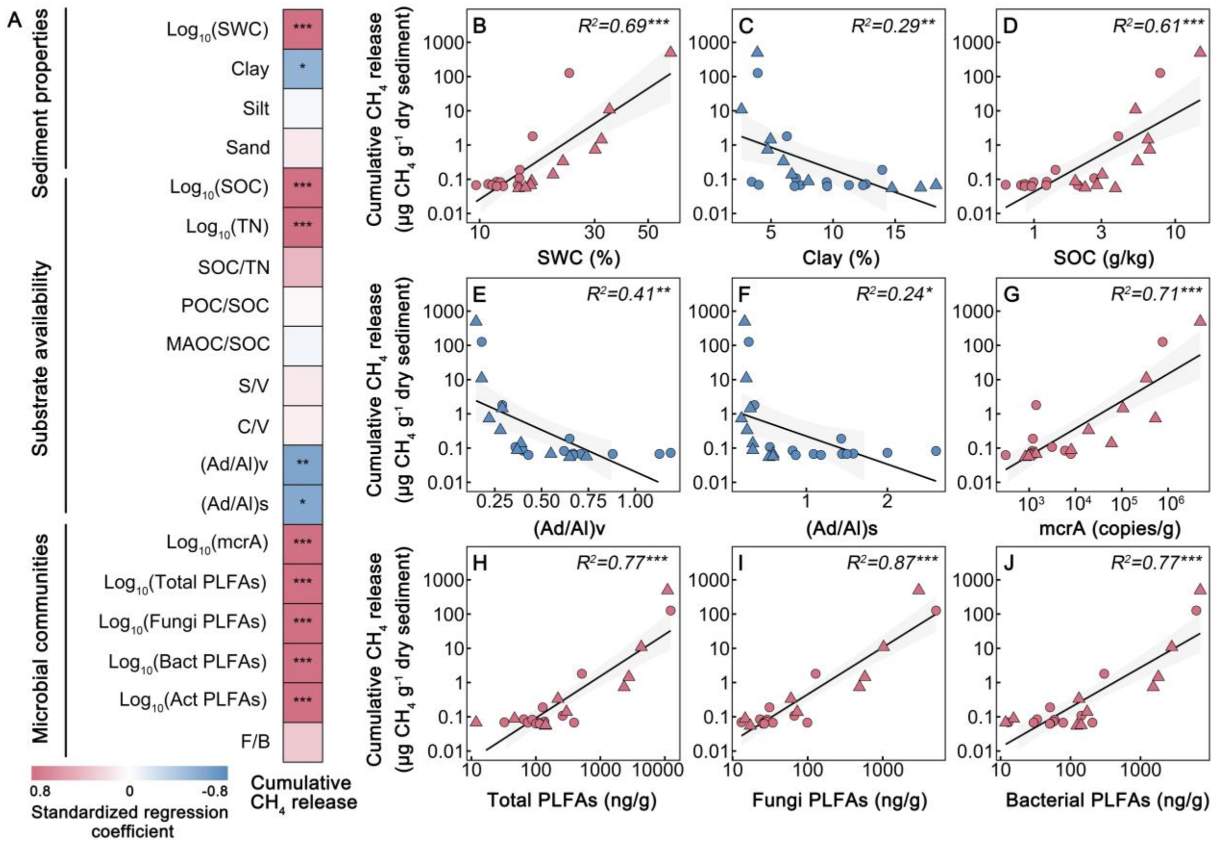

Fig. S7.

Relationships of cumulative CH_4 release with sediment properties, substrate availability, and microbial communities. (A) Standardized regression coefficients of biotic and abiotic factors with cumulative CH_4 release. **(B-J)** Relationships between cumulative CH_4 release and significantly correlated sediment properties, substrate availability, and microbial communities. SWC, sediment water content; SOC, soil organic carbon; TN, total nitrogen; POC/SOC, proportion of particulate organic carbon to sediment organic carbon; MAOC/SOC, proportion of mineral-associated organic carbon to sediment organic carbon; Bact, bacterial PLFAs; Act, actinomycetes PLFAs; F/B, the ratio of fungal PLFAs to bacterial PLFAs; S/V, the ratio of vanillyls to syringyls in ligninphenol; C/V, the ratio of vanillyls to cinnamyls in ligninphenol; The ratio of acid to aldehyde forms of vanillyls and syringyls [(Ad/Al)v and (Ad/Al)s]. Red and blue indicate positive and negative relationships, respectively. The solid lines and grey area represent the linear regression lines and the 95% confidence interval, respectively. Circles and triangles denote BLH-A and BLH-B, respectively. R^2, the proportion of variance explained. *, ** and *** indicate significant correlation between CH_4 release and the corresponding variable at p<0.05, p<0.01, p<0.001, respectively. Source data are provided as a Source Data file.

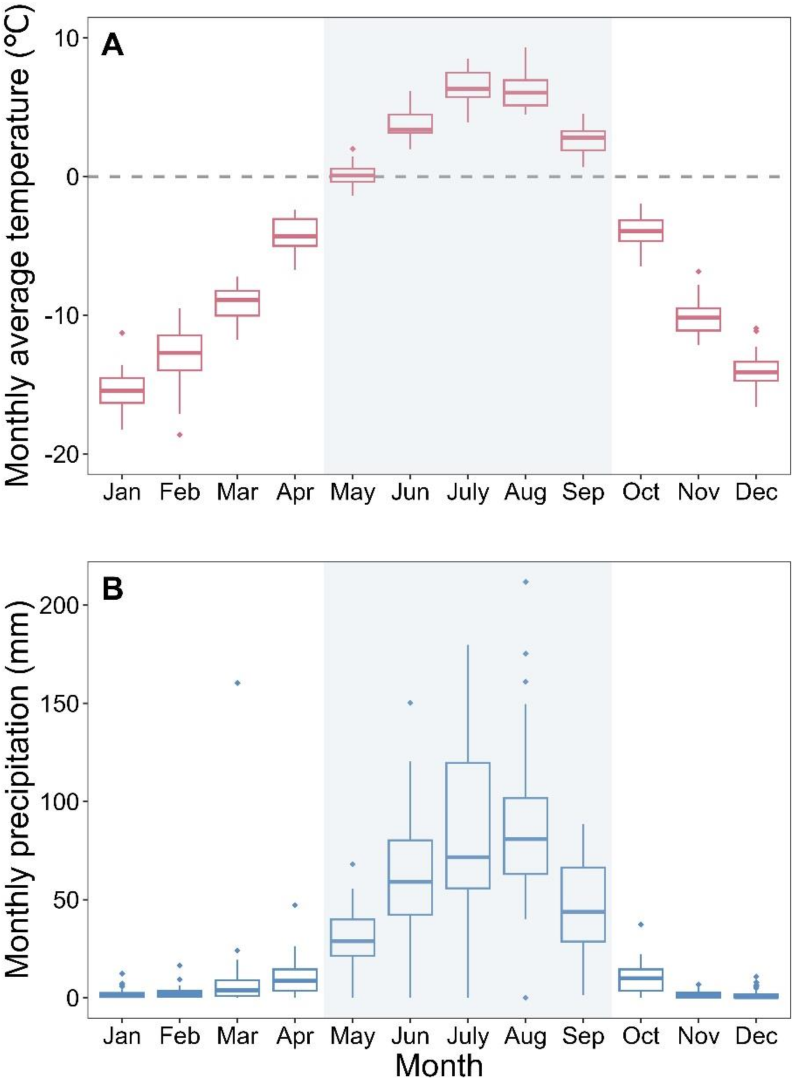

Fig. S8.

Monthly average temperature and precipitation in the study area from the Wudaoliang meteorological station during 1990-2020. (A) Monthly average temperature. **(B)** Monthly precipitation. The solid line and triangle in the box represent the median and mean of each dataset, respectively. The upper and lower ends of boxes denote the 0.25 and 0.75 percentiles, respectively. The upper and lower whisker caps indicate the 1.5 interquartile range of upper and lower quartile, respectively. Dots outside whiskers indicate outliers. Source data are provided as a Source Data file.

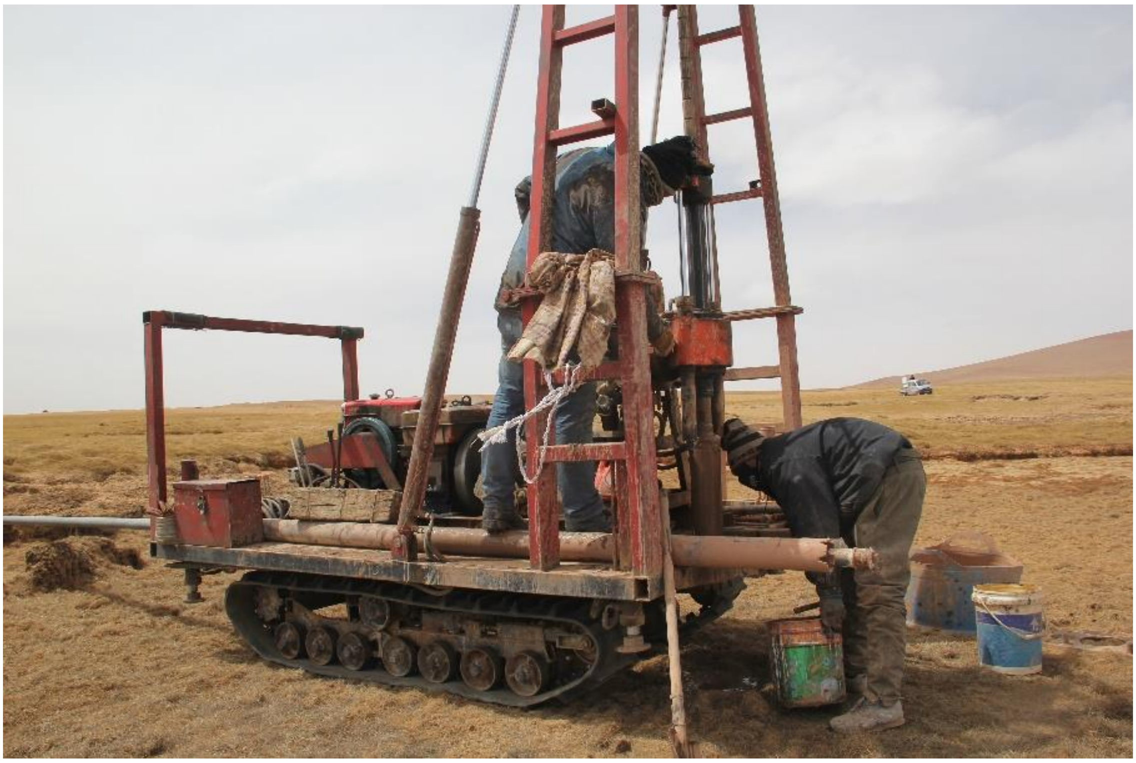

Fig. S9.
Sediment core sampling of drainage-affected thermokarst lakes using a drilling rig. Photographs is taken by Mei Mu.

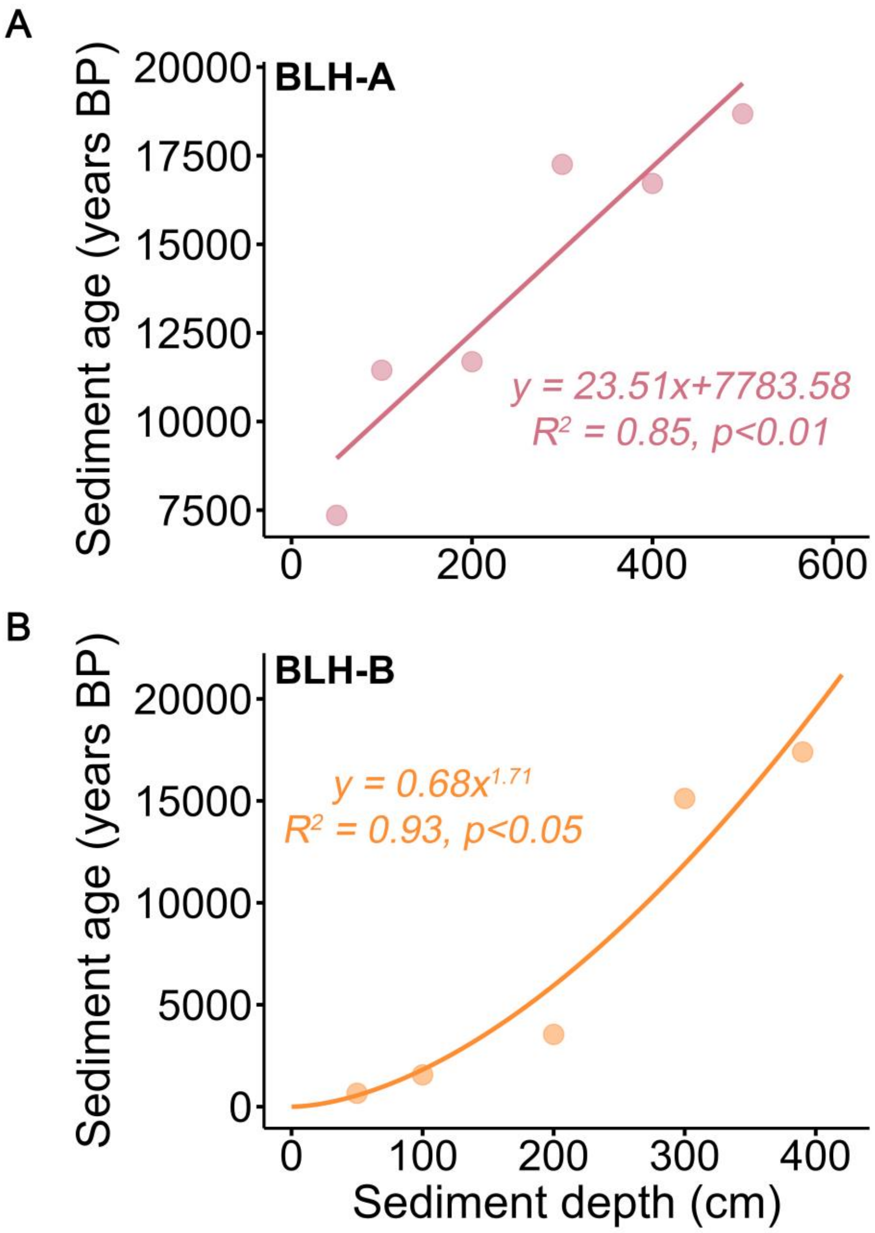

Fig. S10.

The model of age-depth for the thermokarst lake BLH-A (A) and BLH-B (B) .

Source data are provided as a Source Data file.

Table S1.
Characteristics of the sampling sites used in this study.

Lake name	Coordinates	Altitude (m)	Vegetation type	Lake area (m^2)	Lake depth (cm)	Core length (cm)
BLH-A	92.92°E, 34.83°N	4648	Alpine meadow	2530	71	552
BLH-B	92.90°E, 34.83°N	4659	Alpine wet meadow	552	60	387

Table S2

The published and our unpublished Q_{10} data from the non-drainage thermokarst lakes on the QTP.

ID	Lake name	Vegetation type	Sediment depth (cm)	Incubation temp (℃)	Q_{10}	Data source
1	BLH-1	Alpine wet meadow	0-20	5, 15	7.39	Unpublished
2	BLH-2	Alpine wet meadow	0-20	5, 15	1.27	Unpublished
3	BLH-4	Alpine meadow	0-20	5, 15	7.07	Unpublished
4	BLH-7	Alpine meadow	0-20	5, 15	12.77	Unpublished
5	BLH-11	Alpine wet meadow	0-20	5, 15	2.26	Unpublished
6	BLH-12	Alpine wet meadow	0-20	5, 15	6.68	Unpublished
7	BLH-13	Alpine meadow	0-20	5, 15	3.72	Unpublished
8	BLH-15	Alpine meadow	0-20	5, 15	3.83	Unpublished
9	BLH-18	Alpine wet meadow	0-20	5, 15	5.51	Unpublished
10		Alpine wet meadow	0-10	5, 20	21.85	Xu et al., 2024
11		Alpine meadow	0-10	5, 20	6.82	Xu et al., 2024
